# Crosstalk Between Lysine Lactylation and Acetylation Regulates Lactate Dehydrogenase in *Streptococcus mutans*

**DOI:** 10.1093/gpbjnl/qzaf073

**Published:** 2025-08-22

**Authors:** Qizhao Ma, Tao Hu, Yongwang Lin, Jing Li, Jun Huang, Qiong Zhang, Tao Gong, Xuedong Zhou, Lei Lei, Jing Zou, Yuqing Li

**Affiliations:** State Key Laboratory of Oral Diseases & National Center for Stomatology & National Clinical Research Center for Oral Diseases, West China Hospital of Stomatology, Sichuan University, Chengdu 610041, China; Department of Pediatric Dentistry, West China Hospital of Stomatology, Sichuan University, Chengdu 610041, China; State Key Laboratory of Oral Diseases & National Center for Stomatology & National Clinical Research Center for Oral Diseases, West China Hospital of Stomatology, Sichuan University, Chengdu 610041, China; Department of Preventive Dentistry, West China Hospital of Stomatology, Sichuan University, Chengdu 610041, China; State Key Laboratory of Oral Diseases & National Center for Stomatology & National Clinical Research Center for Oral Diseases, West China Hospital of Stomatology, Sichuan University, Chengdu 610041, China; Department of Operative Dentistry and Endodontics, West China Hospital of Stomatology, Sichuan University, Chengdu 610041, China; State Key Laboratory of Oral Diseases & National Center for Stomatology & National Clinical Research Center for Oral Diseases, West China Hospital of Stomatology, Sichuan University, Chengdu 610041, China; Department of Pediatric Dentistry, West China Hospital of Stomatology, Sichuan University, Chengdu 610041, China; State Key Laboratory of Oral Diseases & National Center for Stomatology & National Clinical Research Center for Oral Diseases, West China Hospital of Stomatology, Sichuan University, Chengdu 610041, China; Department of Pediatric Dentistry, West China Hospital of Stomatology, Sichuan University, Chengdu 610041, China; State Key Laboratory of Oral Diseases & National Center for Stomatology & National Clinical Research Center for Oral Diseases, West China Hospital of Stomatology, Sichuan University, Chengdu 610041, China; Department of Pediatric Dentistry, West China Hospital of Stomatology, Sichuan University, Chengdu 610041, China; State Key Laboratory of Oral Diseases & National Center for Stomatology & National Clinical Research Center for Oral Diseases, West China Hospital of Stomatology, Sichuan University, Chengdu 610041, China; State Key Laboratory of Oral Diseases & National Center for Stomatology & National Clinical Research Center for Oral Diseases, West China Hospital of Stomatology, Sichuan University, Chengdu 610041, China; State Key Laboratory of Oral Diseases & National Center for Stomatology & National Clinical Research Center for Oral Diseases, West China Hospital of Stomatology, Sichuan University, Chengdu 610041, China; Department of Preventive Dentistry, West China Hospital of Stomatology, Sichuan University, Chengdu 610041, China; State Key Laboratory of Oral Diseases & National Center for Stomatology & National Clinical Research Center for Oral Diseases, West China Hospital of Stomatology, Sichuan University, Chengdu 610041, China; Department of Pediatric Dentistry, West China Hospital of Stomatology, Sichuan University, Chengdu 610041, China; State Key Laboratory of Oral Diseases & National Center for Stomatology & National Clinical Research Center for Oral Diseases, West China Hospital of Stomatology, Sichuan University, Chengdu 610041, China

**Keywords:** Lysine lactylation, Lysine acetylation, Glycolysis, Lactate dehydrogenase, *Streptococcus mutans*

## Abstract

Post-translational modifications (PTMs) provide essential fine-tuning of protein functions in response to environmental changes. Among the PTMs, lysine acetylation (Kac) and the recently identified lysine lactylation (Kla) play crucial roles in metabolic regulation, as lactate and acetyl-CoA (Ac-CoA) are generated from pyruvate at the end of glycolysis. However, their crosstalk and regulatory mechanisms remain largely unknown, particularly in prokaryotes. Here, we investigated the intricate interrelation between Kla and Kac in the cariogenic bacterium *Streptococcus mutans*, a prolific producer of lactate. We conducted a comprehensive profiling of Kla and Kac, revealing their widespread distribution in glycolytic enzymes. Lactate dehydrogenase (LDH), the terminal enzyme of glycolysis, exhibited dynamic Kla and Kac shifts in line with glycolytic intermediates, with the Kla/Kac ratio reflecting the metabolic influx. Furthermore, ActA was pinpointed as a dual-function acyltransferase that catalyzes the Kla and Kac of LDH, both of which negatively regulate its enzymatic activity. Importantly, the study identified lysine 307 (K307) on LDH as a critical site, with its acylation significantly altering LDH activity, thereby affecting lactate production and bacterial growth. Our insights into the metabolic regulation mediated by Kla and Kac contribute to understanding the metabolism-PTM-metabolism feedback loop, allowing bacteria to fine-tune their metabolism in response to the availability of metabolic intermediates.

## Graphical abstract

**Figure:**
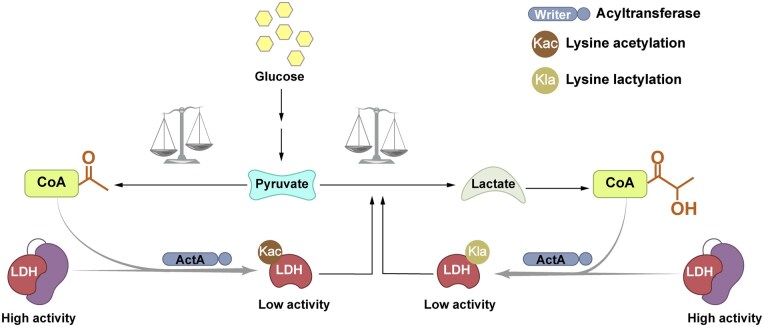

## Introduction

Post-translational modifications (PTMs) are essential processes that occur during or after protein synthesis in which various chemical groups or molecules are covalently attached to proteins [1–3]. The vast array of PTMs and their combinations enable immense diversity and functionality of proteins within a relatively limited number of genes [4]. Among the different types of amino acid residues in proteins, lysine (K) is one of the most common and frequently targeted sites for PTMs [5]. Several PTMs can occur at lysine residues, including acetylation, lactylation, methylation, ubiquitination, succinylation, propionylation, malonylation, butyrylation, glycation, glutarylation, and more [6–11]. These modifications play pivotal roles in maintaining cellular homeostasis and responding to environmental stimuli by fine-tuning the functions of proteins [12].

Protein acylation is a dynamic process influenced by both cellular intrinsic metabolic reprogramming and extrinsic metabolic conditions within the microenvironment [13,14]. For example, both acetyl-CoA (Ac-CoA) and lactyl-CoA (La-CoA), which are generated from pyruvate, the end product of glycolysis, serve as acyl donors for lysine acetylation and lactylation by enzymatic reactions with specific acyltransferases or non-enzymatic reactions; the ratio of these reactions denotes the outlet of pyruvate [15,16]. These metabolic pathways determine the availability of Ac-CoA and La-CoA within the cell, which in turn affects the extent of protein acetylation and lactylation [17,18]. Understanding the connections between cellular metabolism and protein acylation can provide valuable insights into how cells sense and respond to changes in their environment.

Traditionally considered a glycolysis byproduct, lactate is now recognized for its pleiotropic roles in cellular processes, particularly in metabolic reprogramming and protein lactylation, which was first identified in eukaryotic cells [9,19–22]. Regarding prokaryotes, limited knowledge exists about the regulatory mechanisms underlying lysine lactylation. *Streptococcus mutans*, which can prolifically produce lactate under both aerobic and anaerobic conditions, serves as an exemplary candidate to explore the regulatory mechanisms of lactylation in prokaryotes [23,24].

*S. mutans* is well-known for its role in dental caries because it thrives in sugar-rich environments and metabolizes carbohydrates into lactate, which contributes to the acidic environment that promotes tooth decay [25,26]. Previous studies have demonstrated that acetylation is involved in various cellular processes, including the formation of biofilm, metabolism, and stress response, in *S. mutans* [27–29]. Given its prolific production of lactate, the lactylation and acetylation, which are modulated by the availability of La-CoA and Ac-CoA from reprogrammed metabolic pathways, may be an indispensable regulatory mechanism in *S. mutans.* Nevertheless, the understanding of potential crosstalk between lactylation and acetylation remains at an early stage in both eukaryotes and prokaryotes. Owing to the robust ability of *S. mutans* to ferment carbohydrates to produce lactate, it offers a unique opportunity to explore the dynamic and fine-tuned regulation of lactylation and acetylation across changing metabolic states and their potential roles in bacterial pathogenesis and survival.

Here, we profiled the Kla and Kac proteome of *S. mutans* UA159 by merging immunoaffinity enrichment techniques with liquid chromatography-mass spectrometry/mass spectrometry (LC-MS/MS), analyzed the common lysine sites of lactylation and acetylation, and illustrated their crosstalk on bacterial metabolic pathways, primarily the process of glycolysis, which converts glucose into pyruvate by a series of enzymatically catalyzed reactions. We revealed that lactate dehydrogenase (LDH), the terminal enzyme in the glycolysis process that is responsible for the conversion of pyruvate to lactate, undergoes both lactylation and acetylation, which change dynamically with metabolic intermediates during glycolysis. Furthermore, we identified ActA as an acyltransferase that catalyzes both the lysine lactylation and acetylation of LDH, which was found on the same eight lysine side chains. Next, by constructing, expressing, and purifying recombination protein LDH in which lysine (K) was mutated to either glutamine (Q) or arginine (R), we revealed K307 as the major lysine site that is responsible for lactylation and acetylation in LDH. We further demonstrated that LDH K307Q inhibits the activity of LDH and the growth of *S. mutans*, while LDH K307R enhances the activity of LDH and the growth of *S. mutans.* In summary, this study profiles the endogenous proteins with lactylation and acetylation, identifies the acyltransferases for Kla and Kac in LDH, and illustrates the mechanism of metabolic feedback between metabolism and protein acylation.

## Results

### Detection of lysine lactylation and acetylation in *S. mutans*

In our previous study, Kac was identified as a significant regulatory mechanism that influences various physiological processes in *S. mutans* [30]. However, it remains unclear whether the novel Kla exists in *S. mutans*. To determine this, we performed a Western blotting assay with pan-Kla and pan-Kac antibodies on whole-cell lysates from both *S. mutans* and *Escherichia coli*. Owing to recent studies confirming the presence of Kla in *E. coli*, it serves as a positive control to ensure that the method used to detect Kla was functioning correctly [30]. As shown in Figure S1, abundant proteins with lysine residues that were lactylated and acetylated were detected, which confirms the existence of Kla and Kac in *S. mutans*.

### Systematic profiling of the lysine lactylome and acetylome in *S. mutans*

**Figure 1:**
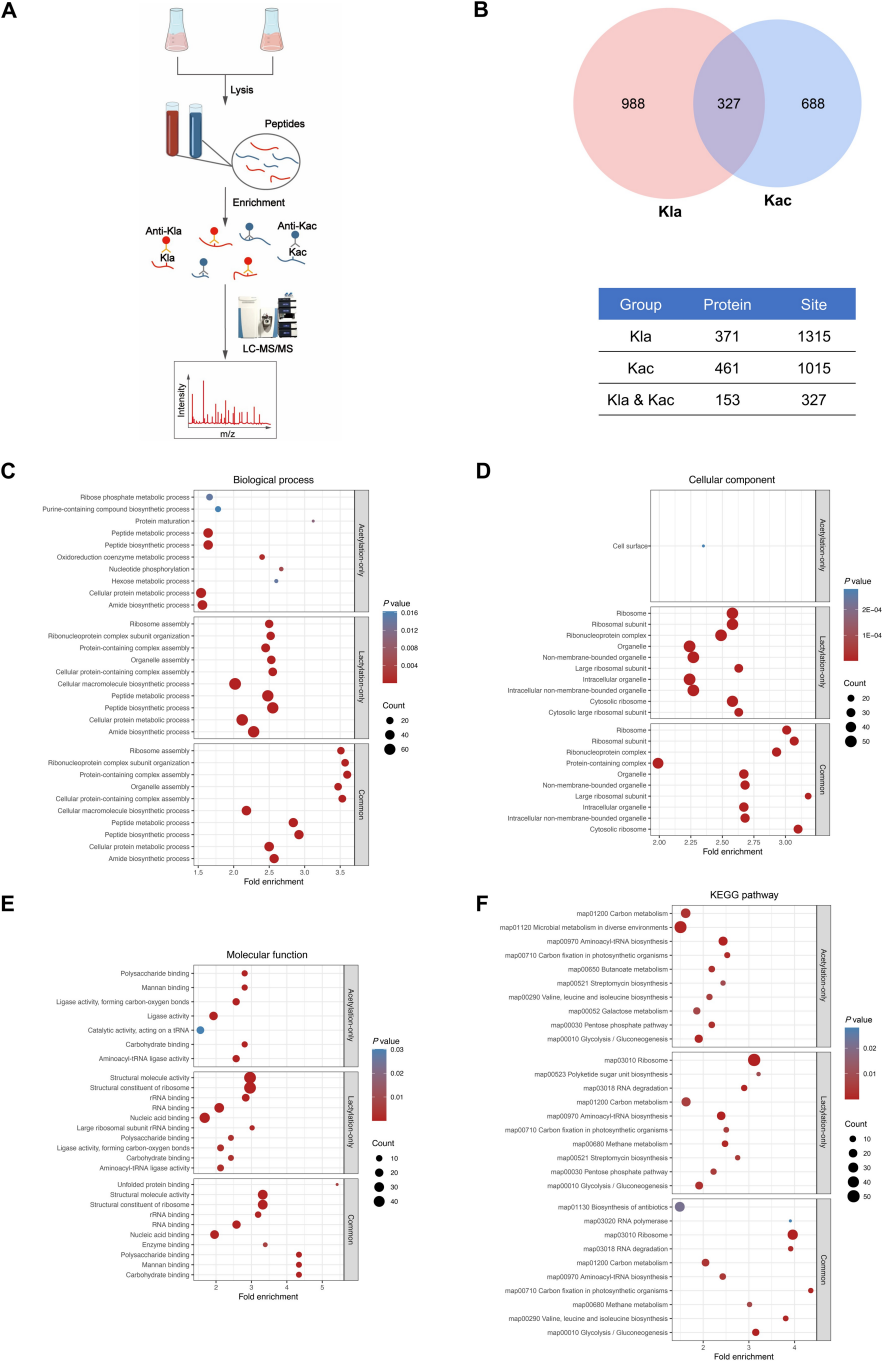
Comprehensively profiling endogenous proteins for Kla and Kac in *S. mutans* **A**. Schematic representation of the experimental workflow for the identification of lactylated and acetylated peptides in *S. mutans*. **B**. Venn diagram depicting the number of proteins and their lysine sites identified to be lactylated and/or acetylated. **C**.–**E**. GO enrichment analysis illustrating the biological processes (C), cellular components (D), and molecular functions (E) associated with the proteins uniquely or commonly lactylated and acetylated. **F**. KEGG pathway analysis showing the pathways enriched among proteins with unique and common lactylation and acetylation lysine sites. Kla, lysine lactylation; Kac, lysine acetylation; GO, Gene Ontology; KEGG, Kyoto Encyclopedia of Genes and Genomes.

To acquire comprehensive insights into the regulation of lactylation and acetylation in *S. mutans*, lysine lactylome and acetylome analyses were performed by culturing cells in Brain Heart Infusion (BHI) media. The cells were harvested at the mid-log phase, and the proteins were extracted for trypsin digestion. The lactylated and acetylated peptides were then enriched by affinity purification using pan-Kla and pan-Kac antibodies and subjected to LC-MS/MS analysis with a high-accuracy mass spectrometer. An overview of the experimental procedures is shown in **Figure 1**A.

A total of 1315 lysine lactylation sites from 371 proteins and 1015 lysine acetylation sites from 461 proteins were identified (Figure 1B; Table S1). Among them, 327 lysine sites from 153 proteins were subjected to both lactylation and acetylation modifications (Figure 1B; Table S1). The substantial overlap between Kla and Kac indicates the potential interaction in their functional roles.

To better understand the biological significance and interaction of Kla and Kac, we further performed Gene Ontology (GO) and Kyoto Encyclopedia of Genes and Genomes (KEGG) pathway analyses of the Kla and Kac proteins with common or unique lysine sites. The GO enrichment analysis showed that the Kla and Kac proteins with common lysine sites, as well as those with unique lysine sites, were mainly related to metabolic and biosynthetic processes and mainly occurred in the ribosome and cytosol with diverse binding activities (Figure 1C–E; Table S2). The KEGG pathway analysis revealed that the Kla and Kac proteins with common lysine sites were enriched in metabolic and biosynthetic pathways, particularly glycolysis/gluconeogenesis, as well as in ribosome, RNA polymerase, and degradation-related pathways (Figure 1F; Table S3). In addition, proteins with exclusive Kla or Kac sites revealed analogous findings (Figure 1F; Table S3). Taken together, the results highlight the crucial preference of Kla and Kac within specific proteins, which may serve as significant regulatory mechanisms in the functional biology of *S. mutans*, particularly in its metabolic and biosynthetic processes.

### Crosstalk analysis between Kla and Kac in *S. mutans*

**Figure 2:**
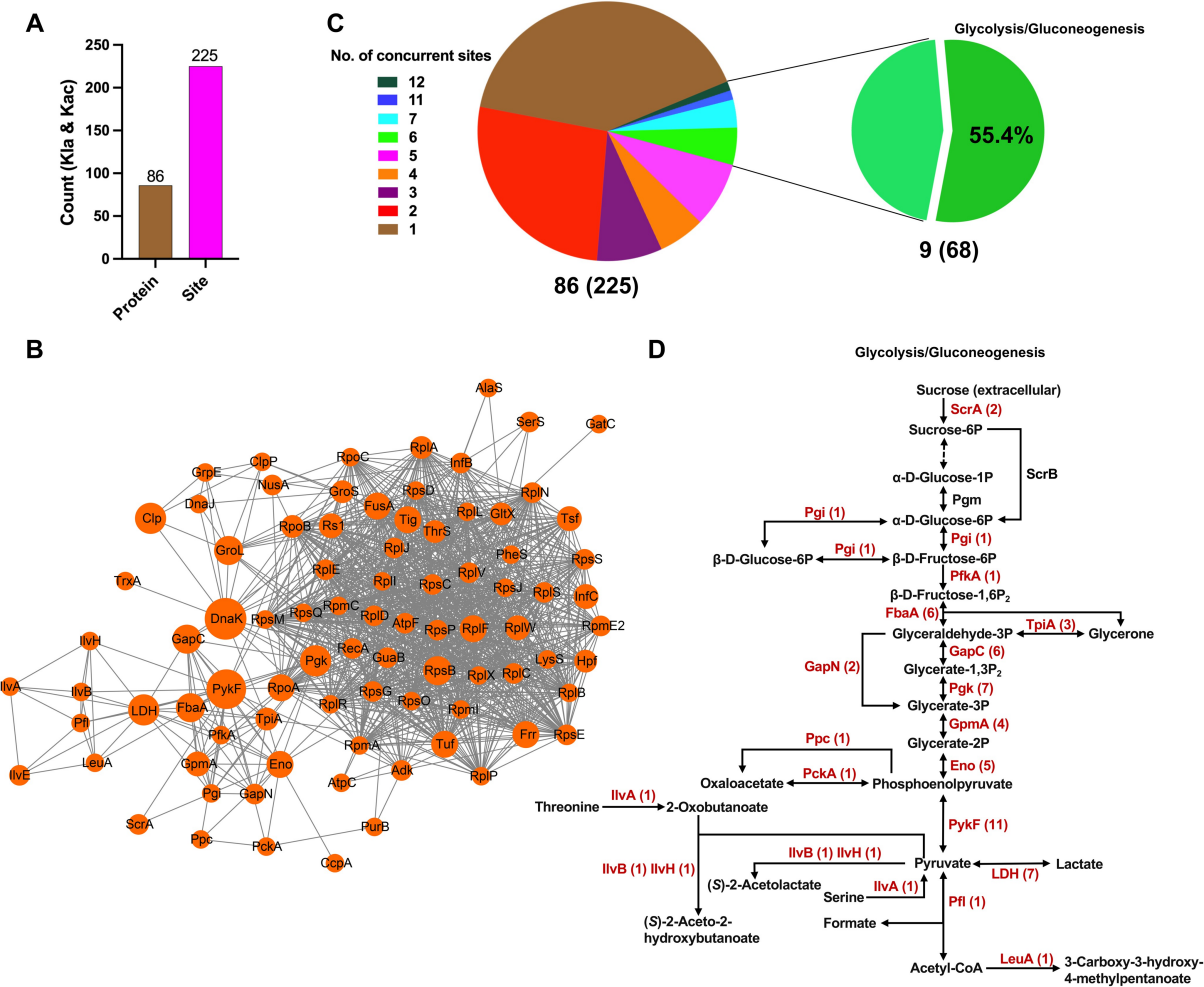
Characterization of the crosstalk between Kla and Kac in *S. mutans* **A**. Statistical analysis of the Kla and Kac proteins and sites of *S. mutans*. **B**. PPINs of the proteins with common Kla and Kac sites, analyzed using the MCODE plug-in toolkit in the Cytoscape software. **C**. Distribution of the concurrent Kla and Kac sites across proteins. A total of 86 proteins (225 lysine sites) were found to harbor both Kla and Kac modifications, with 9 proteins (68 lysine sites) showing more than six concurrent Kla and Kac sites. **D**. Enrichment of proteins with common Kla and Kac sites in the glycolysis/gluconeogenesis pathway. The biacylated protein and their lysine site numbers are marked in red. MCODE, Molecular Complex Detection; PPINs, protein-protein interaction networks.

To gain insight into the potential crosstalk between Kla and Kac in the metabolic and biosynthetic processes of *S. mutans*, protein–protein interactions (PPIs) and PPI networks (PPINs) were constructed and analyzed. As shown in **Figure 2**A and B; Table S4, there were 86 proteins with 225 common Kla and Kac sites as nodes, which exhibited 1022 direct physical interactions in metabolism, translation, ribosome assembly, and biosynthesis. An analysis of the distribution of these concurrent Kla and Kac sites across proteins showed that most (∼ 67.4%) of the identified proteins possessed one to two lysine sites, and 10.5% had more than six coexisting Kla and Kac sites. Interestingly, when examining the proteins with more than six coexisting Kla and Kac sites, we found that 68 sites were associated with nine proteins (Figure 2C). To explore the biological significance of these proteins, we conducted a PPI network analysis and a KEGG pathway enrichment analysis. The PPI network analysis highlighted key interactions between these proteins, reinforcing their functional relevance in cellular processes (Figure S2A). Furthermore, KEGG analyses revealed that the majority of these proteins, accounting for 54.4% of the total modification sites with more than six coexisting Kla and Kac sites, are enriched in the glycolysis/gluconeogenesis pathway, highlighting the potential role of Kla and Kac modifications in regulating key steps in bacterial glycolysis/gluconeogenesis (Figure S2B).

To better understand the roles of Kla and Kac, we mapped the proteins with Kla and Kac to KEGG pathways based on lysine lactylome and acetylome results from three biological replicates. As shown in Figure 2D, nearly all the enzymes in the glycolysis/gluconeogenesis pathway were both lactylated and acetylated. It is worth noting that when the enzymes were closer to the terminal stages of glycolysis/gluconeogenesis, there were more sites where both Kla and Kac occurred, suggesting the potential significance of their roles in the fine-tuning and efficient regulation of the metabolic processes described above. Taken together, our analyses indicated that there was extensive existence of Kla and Kac on the enzymes at the same sites involved in the glycolysis/gluconeogenesis pathways.

### Dynamic regulation of Kla and Kac of LDH during glycolysis

As shown in Figure 2D, seven lysine sites in LDH that are responsible for the production of lactate from pyruvate were both lactylated and acetylated in *S. mutans*. A recent study by Zhang showed that lactate is a critical determinant of protein Kla, suggesting that the amount of lactate available inside the cell can directly influence the extent of protein Kla. Given the significance of LDH in the glycolytic pathway and cellular metabolism, understanding the regulatory mechanisms underlying the crosstalk between Kla and Kac on LDH can offer insights into how the activity of LDH is fine-tuned in response to cellular conditions and how the glycolysis pathway is dynamically regulated by Kla and Kac.

**Figure 3:**
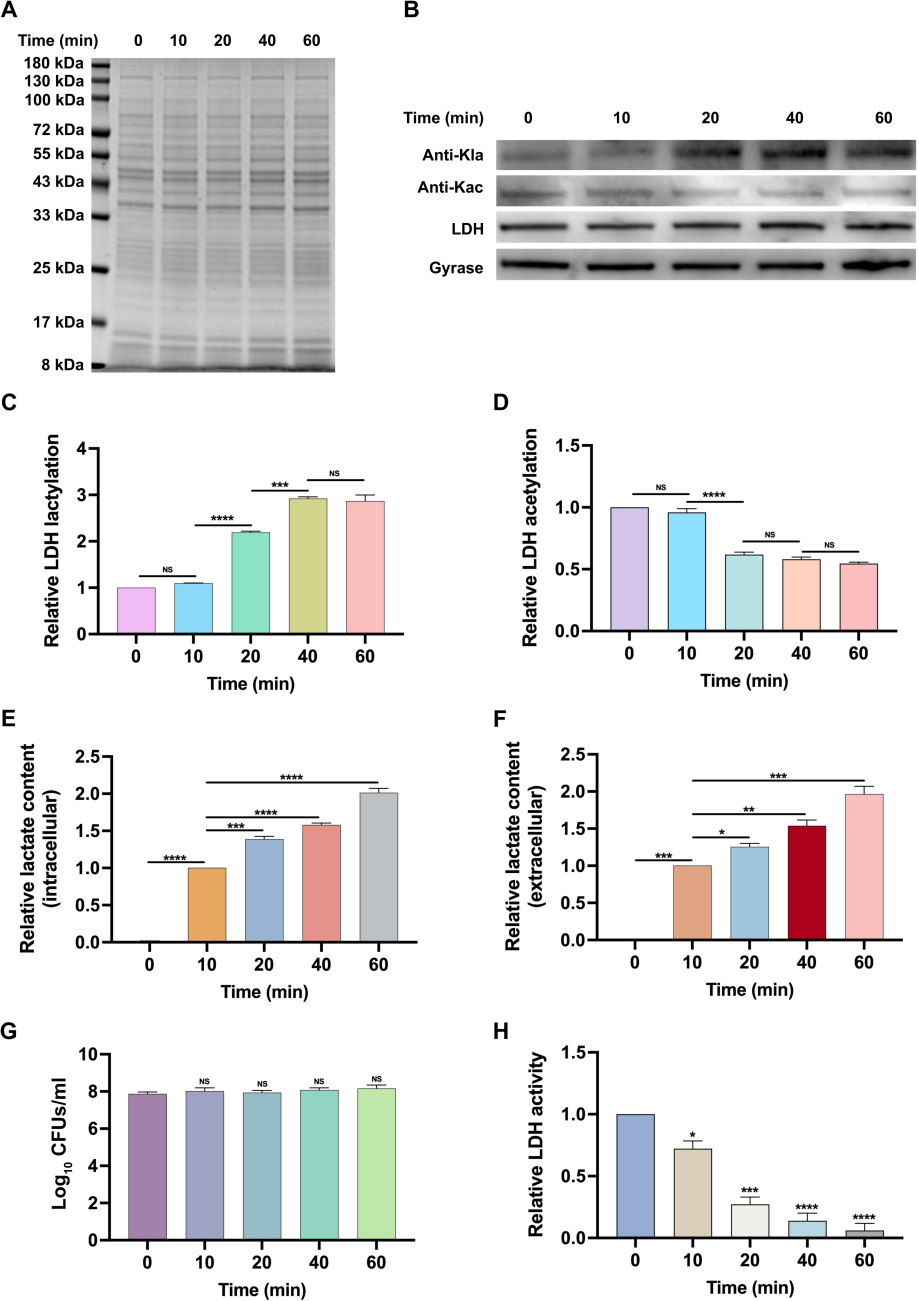
Temporal changes in LDH Kla and Kac, lactate production, and LDH activity during glycolysis *S. mutans* UA159 was grown in the presence of 1% glucose to trigger glycolysis. The following parameters were analyzed at different intervals: the intracellular protein levels (**A**), intracellular LDH Kla and Kac levels (**B**–**D**), intracellular (**E**) and extracellular (**F**) lactate production, CFUs (**G**), and LDH activity (**H**). The results are presented as mean ± SD. Statistical significance was determined using one-way ANOVA (*, *P* < 0.05; **, *P* < 0.01; ***, *P* < 0.001; ****, *P* < 0.001; NS, not significant). LDH, lactate dehydrogenase; SD, standard deviation; ANOVA, analysis of variance; GFUs, colony forming units.

To examine whether the level of lactate affects the Kla and Kac of LDH, we evaluated the intracellular and extracellular concentrations of lactate and the corresponding Kla and Kac levels of LDH in a glycolytic pH drop assay (Figure S3). As glycolysis proceeded, there was a significant increase in the level of Kla in LDH, which stabilized after 40 min of glucose exposure, whereas the level of Kac in LDH significantly decreased and reached a stable state after 20 min of glucose exposure (**Figure 3**A–D). Moreover, to determine whether the upregulated Kla level of LDH is associated with the level of lactate, we assessed both the intracellular and extracellular production of lactate. As shown in Figure 3E and F, the levels of both intracellular and extracellular lactate increased significantly as the glycolysis proceeded for 60 min. Furthermore, the colony forming units (CFUs) were measured to exclude the possibility that the observed decrease in the intracellular production of lactate was owing to variation in the total bacterial counts. There were no significant differences in the CFUs among *S. mutans* samples that were collected from different intervals of glycolysis (Figure 3G).

Given the potential impact of lysine acylation on the functionality of enzymes, we explored the effects of Kla and Kac on the activity of LDH. The results showed that the activity of LDH was significantly inhibited compared with its activity at the beginning of glycolysis over 60 min (Figure 3H). These results suggest that lactate dynamically affects the Kla and Kac of LDH, which in turn regulate its enzymatic activity and ultimately influence the production of lactate.

### ActA mediates the Kla and Kac of LDH to regulate its enzymatic activity

Protein acylation, which can be regulated non-enzymatically or enzymatically, modulates protein functions in diverse cellular processes [31,32]. Therefore, identifying the enzymes that regulate Kla and Kac becomes vital to comprehending their roles in *S. mutans*. The lysine acetyltransferase (KATs) family was first regarded as the writers of acetylation. In contrast, with the discovery of novel types of protein acylation, many members of the KAT family were shown to have an expanded repertoire of other short-chain acyltransferase activities [33–40]. Our previous studies have shown that acetyltransferase ActA regulates the acetylation of lysine in LDH in *S. mutans* [27]. Therefore, we hypothesized that ActA could also be responsible for the lactylation of lysine in LDH in *S. mutans*.

**Figure 4:**
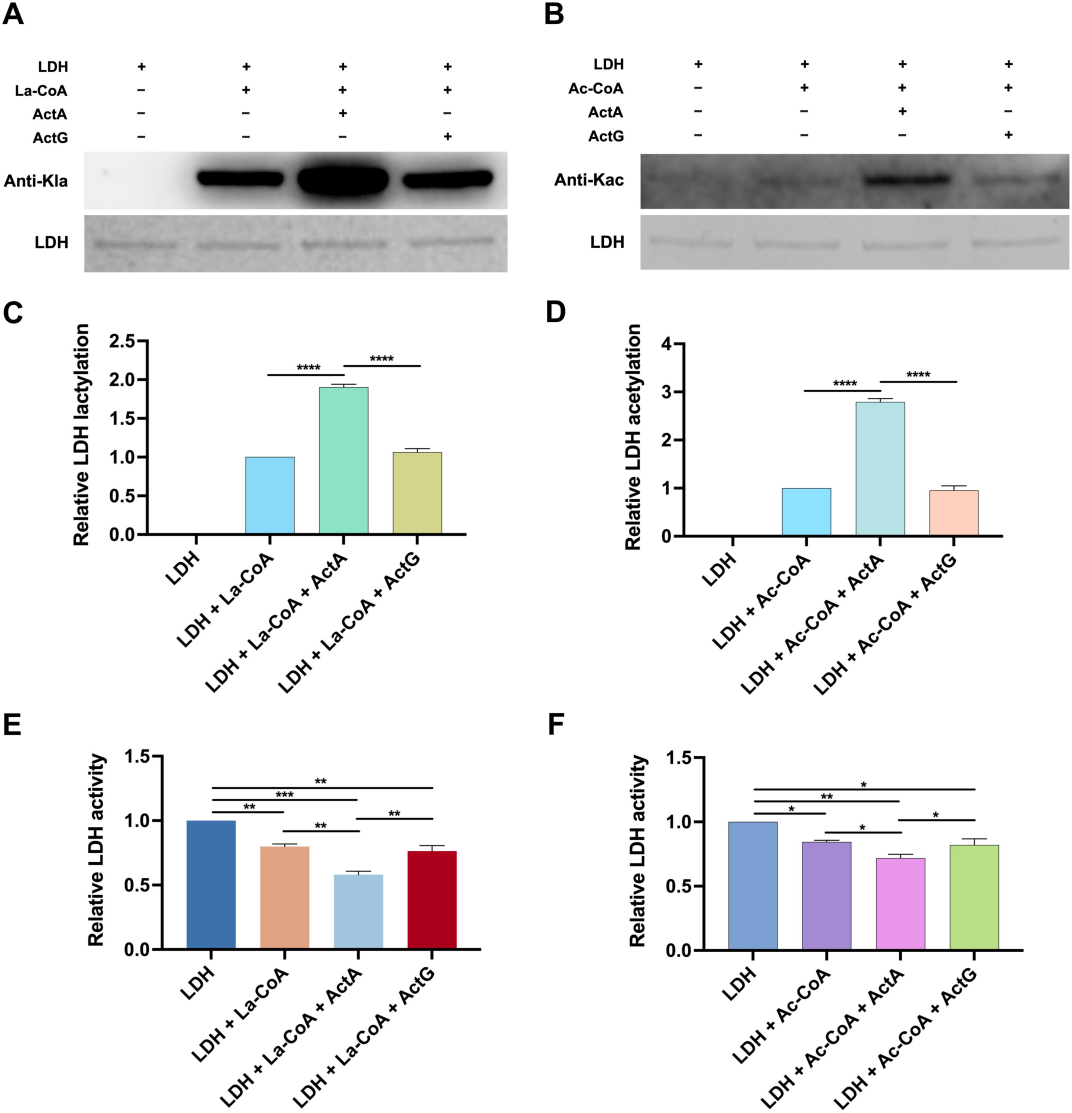
ActA mediates the Kla and Kac of LDH and regulates its enzymatic activity Recombinant LDH was incubated with ActA or ActG in the presence or absence of La-CoA and Ac-CoA for 3 h at 37°C. **A**. and **B**. Western blotting of LDH lactylation in the presence of La-CoA (A) and LDH acetylation in the presence of Ac-CoA (B). **C**. and **D**. Quantification of anti-acyl bands with ImageJ software for Kla (C) and Kac (D). **E**. and **F**. Analysis of LDH activity in the La-CoA-treated group (E) and the Ac-CoA-treated group (F). Data are presented as mean ± SD. Statistical significance was determined using one-way ANOVA for multiple comparisons, or Student’s *t*-test for comparisons between two groups. *, *P* < 0.05; **, *P* < 0.01; ***, *P* < 0.001; ****, *P* < 0.001. La-CoA, lactyl-CoA; Ac-CoA, acetyl-CoA.

Firstly, we examined the intracellular Kla and Kac levels of LDH in the control strain UA159/pDL278 and the *actA* overexpression strain UA159/pDL278-*actA*. As shown in Figure S4, *actA* overexpression significantly increased the Kla and Kac levels of LDH in *S. mutans*. To further demonstrate the role of ActA as an acyltransferase for LDH lactylation, ActA and LDH were cloned, expressed, and purified from *E. coli* (Figure S5), as well as ActG, another known acetyltransferase in *S. mutans*, which is involved in the acetylation of glucosyltransferases (Gtfs). ActG was selected as the negative control to assess the specific effect of ActA on acylating LDH. The *in vitro* acylation assays of LDH were then conducted where ActA and ActG served as the acyltransferases, La-CoA and Ac-CoA as the acyl group donors, and LDH as the substrate. As shown in **Figure 4**A–D, when LDH was solely exposed to La-CoA and Ac-CoA, both induced the Kla and Kac of LDH non-enzymatically. However, ActA significantly increased the levels of Kla and Kac in LDH enzymatically. As expected, ActG did not exert a significant effect on the levels of Kla and Kac in LDH.

Furthermore, we evaluated the effects of Kla and Kac on the enzymatic activities of LDH. Compared with the unmodified LDH, the Kla and Kac of LDH both significantly inhibited its enzymatic activities (Figure 4E and F). Altogether, these results indicate that ActA functions as an endogenous acyltransferase involved in the Kla and Kac of LDH, both of which negatively regulate its enzymatic activities.

### Impact of metabolic intermediates on the Kla and Kac of LDH

**Figure 5:**
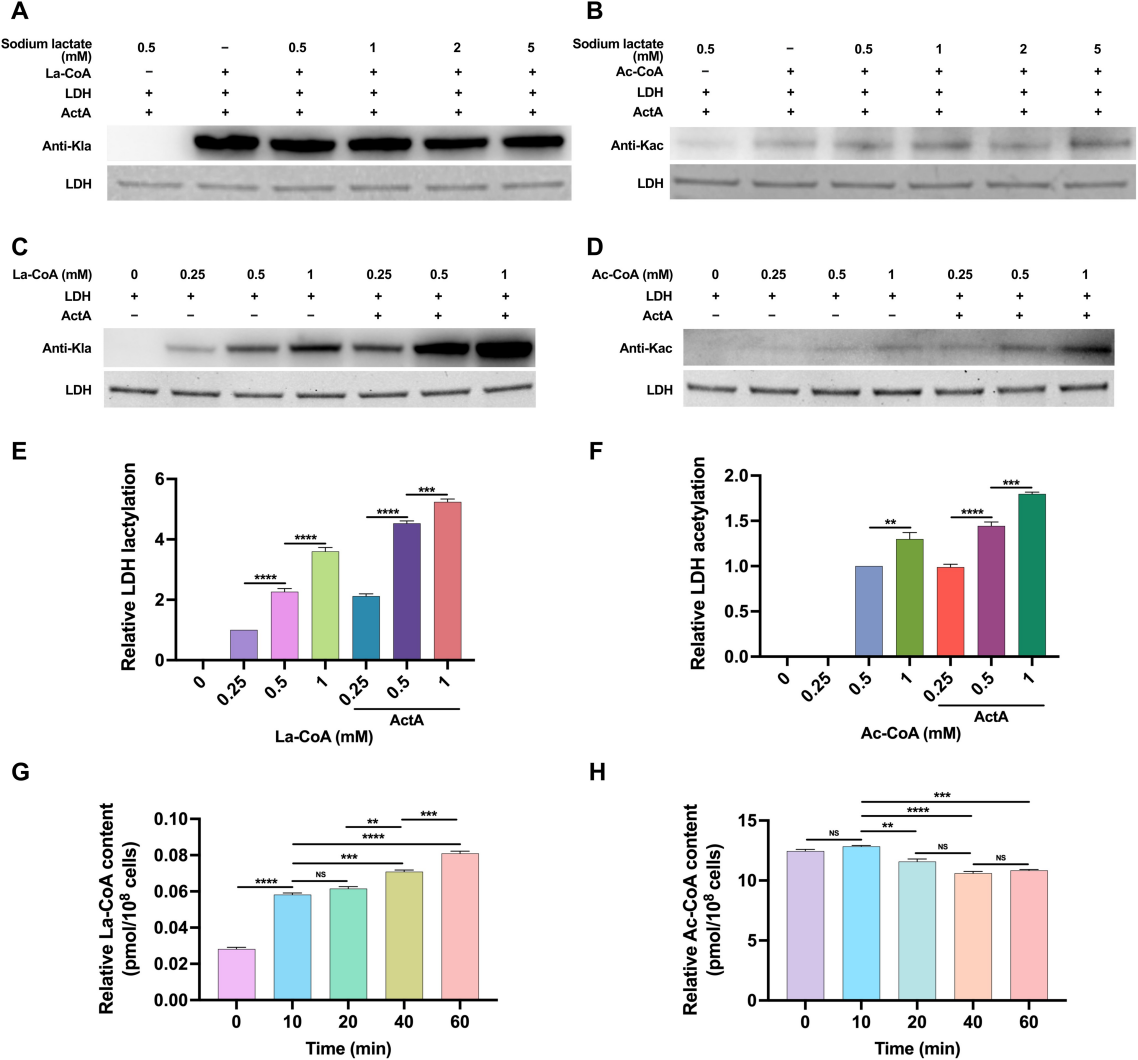
Effects of metabolic intermediates on the Kla and Kac of LDH **A**. and **B**. Effects of different concentrations of sodium lactate on the levels of Kla (A) and Kac (B) of LDH during *in vitro* acylation assays, analyzed by Western blotting. **C**. and **D**. Effects of different concentrations of La-CoA and Ac-CoA on the Kla (C) and Kac (D) levels of LDH during the *in vitro* acylation assays. **E**. and **F**. Quantification of anti-acyl bands with ImageJ software for Kla (E) and Kac (F). **G**. and **H**. Analysis of the intracellular concentrations of La-CoA (G) and Ac-CoA (H) in *S. mutans* UA159 grown in the presence of 1% glucose at different time intervals. Data are presented as mean ± SD. Statistical significance was determined using one-way ANOVA (*, *P* < 0.05; **, *P* < 0.01; ***, *P* < 0.001; ****, *P* < 0.001; NS, not significant).

It has been established that lactylation of lysine correlates positively with the levels of intracellular lactate [41]. However, there was no evidence indicating whether lactate directly affected the protein Kla or its crosstalk with other acylating types *in vitro*. To confirm this, we monitored the Kla and Kac levels of LDH in the *in vitro* acylation reaction mix by adding different concentrations of sodium lactate. No significant differences in the levels of Kla and Kac of LDH were observed irrespective of the concentration of sodium lactate (**Figure 5**A and B). However, we found that lactate dynamically affects the Kla and Kac levels of LDH in the glycolytic pH drop assay (Figure 3A–F). Considering that the metabolic intermediates La-CoA and Ac-CoA can act as acyl group donors for the proteins Kla and Kac, we then measured the effect of their concentrations on the levels of Kla and Kac of LDH in *in vitro* acylation assays. As shown in Figure 5C–F, both La-CoA and Ac-CoA significantly increased the levels of Kla and Kac of LDH, either non-enzymatically or enzymatically, in a dose-dependent manner.

To further understand the dynamic changes in the Kla and Kac of LDH, we explored the concentrations of La-CoA and Ac-CoA in the glycolytic pH drop assay. As expected, the level of La-CoA significantly increased with glycolysis at 10 min, and that of Ac-CoA significantly decreased with glycolysis at 20 min, which was consistent with the observed Kla and Kac levels of LDH (Figure 5G and H). Together, our results demonstrated that the intracellular availability of La-CoA and Ac-CoA, rather than lactate, directly influences the extent of Kla and Kac of proteins.

### Identification of K307 as the predominant functional site for both Kla and Kac in LDH

**Figure 6:**
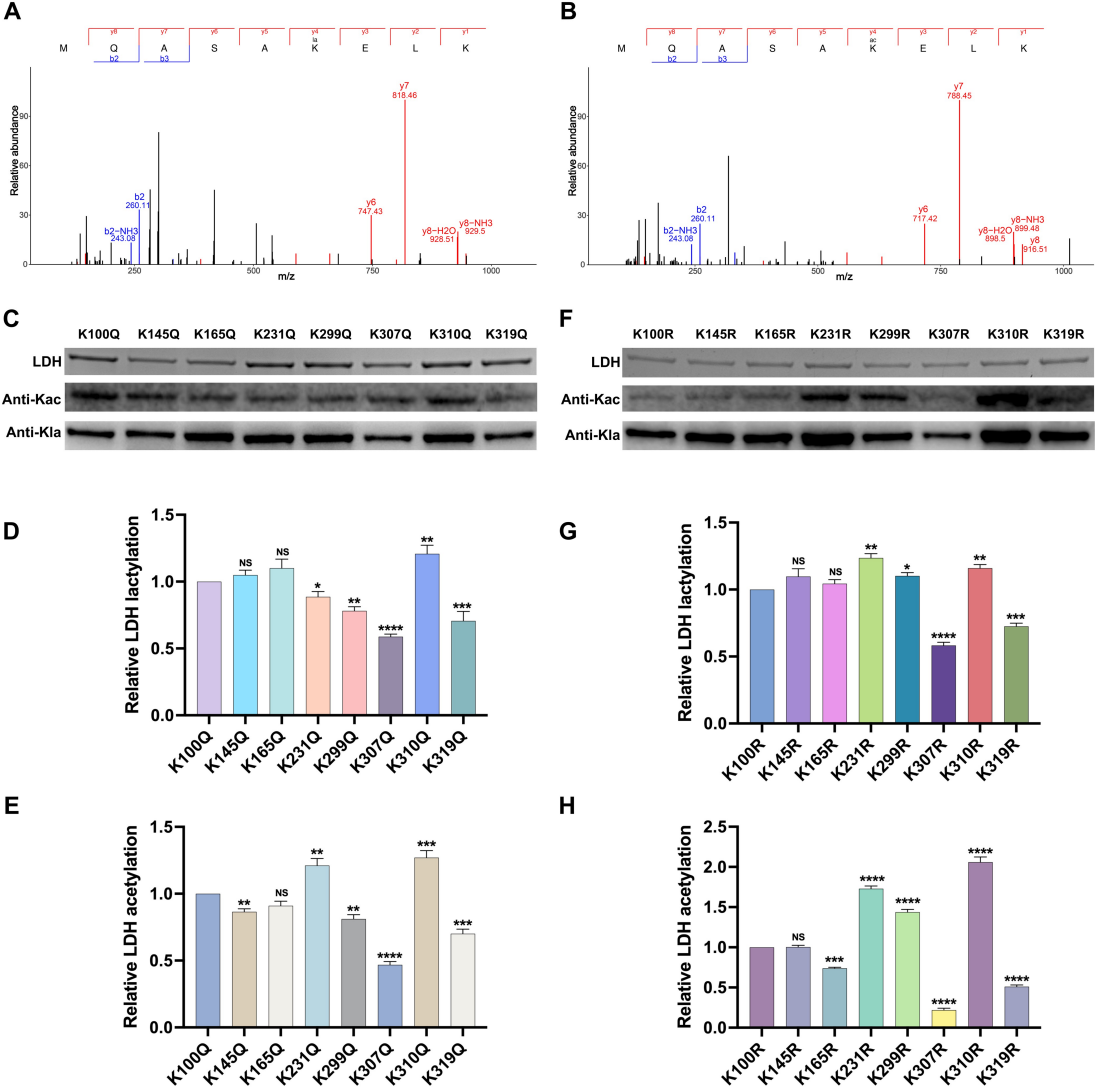
Identification of the predominant lysine site among eight common Kla and Kac sites **A**. and **B**. Representative MS/MS spectra of peptides MQASAK(la)ELK (A) and peptides MQASAK(ac)ELK (B) from eight putative lysine sites identified with common Kla and Kac in LDH. **C**.Western blotting for recombinant LDH with site-directed mutagenesis of these eight lysine sites to glutamine, incubated with ActA in the presence of La-CoA and Ac-CoA. **D**. and **E**. Quantification of anti-acyl bands in (C) using ImageJ software for Kla (D) and Kac (E). **F**. Western blotting for recombinant LDH with site-directed mutagenesis of these eight lysine sites to arginine, incubated with ActA in the presence of La-CoA and Ac-CoA. **G**. and **H**. Quantification of anti-acyl bands in (F) using ImageJ software for Kla (G) and Kac (H). Data are presented as the mean ± SD. Statistical significance was determined using one-way ANOVA (*, *P* < 0.05; **, *P* < 0.01; ***, *P* < 0.001; ****, *P* < 0.001; NS, not significant).

To identify the predominant functional sites of Kla and Kac in LDH, the enzyme was incubated with La-CoA and Ac-CoA, in either the presence or absence of ActA, followed by MS analysis. The results revealed that eight putative lysine sites were both lactylated and acetylated in LDH, which is basically consistent with the identified lysine sites in the lactylome and acetylome (**Figure 6**A and B, Figures S6 and S7; Table S5).

To further understand the significance of these sites, we individually mutated each of the eight lysine sites to either glutamine (Q) and arginine (R). A mutation to glutamine neutralized the positive charge and mimicked the structure of an acylated lysine, whereas a mutation to arginine maintained the positive charge and avoided acylation (Figure S8). The mutation of K307 but not other lysine residues to glutamine resulted in a significant reduction in the levels of both Kla and Kac of LDH (Figure 6C–E). Similarly, the arginine substitution of K307 dramatically decreased the levels of Kla and Kac of LDH (Figure 6F–H). Collectively, these results indicate that K307 is the principal lysine site that mediates both Kla and Kac of LDH in *S. mutans*.

### K307 regulates LDH activity and influences the production of lactate and bacterial growth

**Figure 7:**
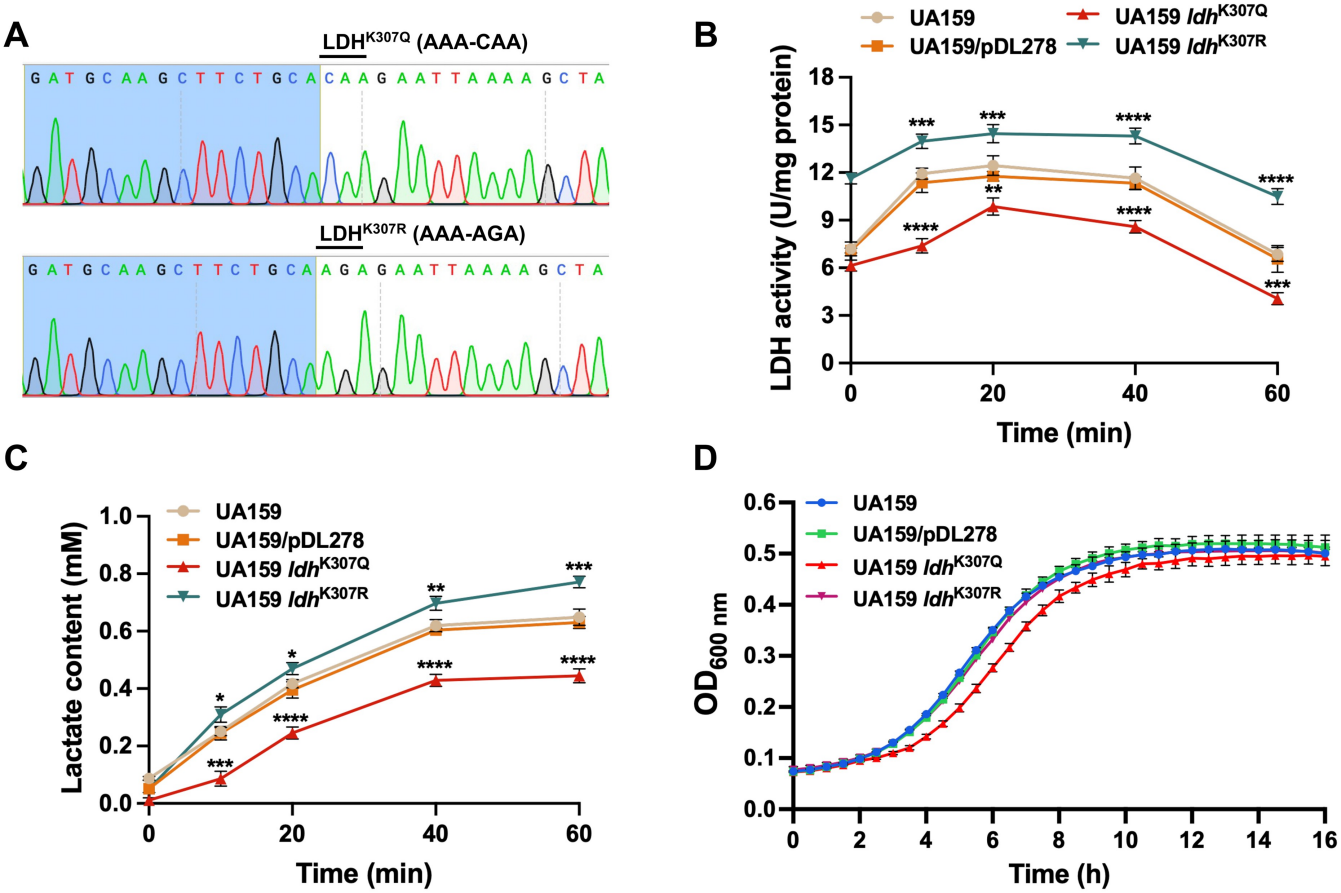
K307 regulates LDH activity and influences the production of lactate and bacterial growth **A**. Confirmation of successful generation of the LDH point mutation (AAA-CAA for LDH^K307Q^ and AAA-AGA for LDH^K307R^) strains UA159 *ldh*^K307Q^ and UA159 *ldh*^K307R^ using the CRISPR-Cas9 technique. **B**. and **C**. LDH activity (B) and lactate production (C) in different *S. mutans* strains. **D**. Growth curves of different *S. mutans* strains cultured in TV medium supplemented with 1% glucose at 37°C in 96-well plates for 16 h. TV, tryptone-vitamin. Data are presented as mean ± SD. Statistical significance was determined using one-way ANOVA (*, *P* < 0.05; **, *P* < 0.01; ***, *P* < 0.001; ****, *P* < 0.001).

To investigate the effect of K307 acylation on LDH function, point mutation (LDH^K307Q^ and LDH^K307R^) strains were constructed in *S. mutans* using the CRISPR-Cas9 technique. The sequencing results showed that the point mutation (AAA-CAA for LDH^K307Q^ and AAA-AGA for LDH^K307R^) strains UA159 *ldh*^K307Q^ and UA159 *ldh*^K307R^ were successfully constructed (**Figure 7**A). We then assayed the intracellular LDH activity in the UA159, UA159/pDL278, UA159 *ldh*^K307Q^, and UA159 *ldh*^K307R^ strains, with the UA159 and UA159/pDL278 serving as controls. As shown in Figure 7B, the UA159 *ldh*^K307Q^ strain had the lowest activity, and the UA159 *ldh*^K307R^ strain exhibited the highest activity among the four strains at the different intervals of the glycolytic process. Consistent with the LDH activity, the UA159 *ldh*^K307Q^ strain produced the least amount of lactate, and the UA159 *ldh*^K307R^ strain had the highest production of lactate among the four strains at the different intervals of the glycolytic process (Figure 7C).

Notably, metabolic and biosynthetic processes affect bacterial growth [42,43]. Therefore, we rationally predicted that the changes in LDH activity induced by Kla and Kac could influence the growth of *S. mutans*. To confirm this hypothesis, considering that LDH catalyzes the conversion of the pyruvate derived from glycolysis to lactate, we selected a tryptone-vitamin (TV) base medium containing 1% (w/v) glucose, allowing LDH to perform its standard metabolic function. The growth curves were measured after culture in TV with 1% glucose. We found that the UA159 *ldh*^K307R^ strain showed no significant differences in growth, and the UA159 *ldh*^K307Q^ strain grew the slowest among the four strains (Figure 7D). Together, these results demonstrate the critical role of K307 acylation in modulating the activity of LDH, which, in turn, affects the production of lactate and bacterial growth.

## Discussion

Pyruvate, the terminal metabolite of glycolytic processes, serves as a key metabolic node in the network of cellular metabolism [44,45]. Under aerobic conditions, pyruvate is further converted to Ac-CoA by the pyruvate dehydrogenase complex to fuel the tricarboxylic acid (TCA) cycle and oxidative phosphorylation for efficient energy production [46]. In the absence of oxygen, more pyruvate is converted to lactate by LDH [47]. These metabolic intermediates generated from pyruvate have been shown to play roles in connecting the cellular metabolism to PTMs [13,48,49]. This includes Ac-CoA, which donates the acetyl group for Kac. However, the PTM of lactate (formerly considered a metabolic waste) on lysine was not identified until 2019 when lactylation was discovered in eukaryotes. Whether there is a crosstalk between Kla and Kac and what biological functions they might perform remains unclear in prokaryotes. Thus, it is necessary to explore the characterization, functions, and dynamic regulatory mechanisms of Kla and Kac in bacteria. In this case, *S. mutans* could serve as a model organism to evaluate the crosstalk between Kla and Kac owing to its strong ability to convert pyruvate to lactate by LDH, regardless of oxygen availability, and its metabolic plasticity that can reduce pyruvate to Ac-CoA by pyruvate-formate lyase (PFL) and the pyruvate dehydrogenase complex (PDH) under anaerobic and aerobic conditions, respectively [50,51].

In this study, we uncovered that Kla and Kac are prevalent in the proteins of *S. mutans*. The results are consistent with recent studies indicating that acetylation and other types of acylation, such as succinylation and crotonylation, coexist on the same protein or even on the same lysine residue [5,52,53]. Interestingly, the proteins with both Kla and Kac were primarily enriched in metabolic and biosynthetic processes. Mapping the PPINs enabled us to further explore the functional interactions of proteins with Kla and Kac, which were particularly closely related to the glycolytic pathway. As shown in Figure 2D, almost all the enzymes in glycolysis were lactylated and acetylated, which are critical for bacterial survival and pathogenicity. Notably, these enzymes closer to the glycolytic endpoint exhibit more lysine sites with both Kla and Kac. Such a preference for Kla and Kac in the glycolytic enzymes suggests a tightly coordinated regulatory mechanism where the levels of Kla and Kac may adapt to changes in the metabolic landscape.

Among the glycolytic enzymes, LDH is unique owing to its pivotal role in maintaining a high rate of glycolysis by regenerating NAD^+^ from the NADH, which is required as a cofactor for the early glycolytic steps [54]. Moreover, lactate, converted by LDH from pyruvate, positively correlates with the levels of protein lactylation [55]. In this study, LDH undergoes both Kla and Kac with dynamic changes depending on the concentration of lactate during glycolysis, which causes a significant reduction in enzymatic activity. Thus, acylation may act as a metabolic feedback mechanism that enables bacteria to fine-tune glycolysis and their production of lactate.

Furthermore, this study identified ActA as an acyltransferase that can catalyze both the Kla and Kac of LDH. Our previous studies have shown that ActA is responsible for the acetylation of LDH in *S. mutans*, broadening the functional repertoire of this enzyme to acylation. This aligns with similar findings, such as the identification of P300 as the common lysine acyltransferase for almost all short-chain protein acylations [56–61]. In particular, both the Kla and Kac of LDH, catalyzed by ActA, lead to a significant decrease in its activity, which suggests the presence of a possible regulatory mechanism that prevents the excessive production of lactate, which could be detrimental to *S. mutans*. The intricate crosstalk between Kla and Kac was further illustrated during *in vitro* and *in vivo* assays, which demonstrated that the concentrations of the acyl group donors, rather than lactate itself, directly influenced the extent of Kla and Kac of LDH. Therefore, this research expanded the understanding of acyltransferases in regulatory and functional diversity. The balance between Kla and Kac depends on the metabolic intermediates of acyl-CoA, which in turn regulate the metabolic processes involving acylated substrates. Under physiological conditions, pyruvate is primarily shunted toward the TCA cycle, where it is converted to Ac-CoA. This metabolic shift subsequently enhances the Kac of proteins since Ac-CoA serves as a donor of acetyl groups. Conversely, at a high rate of glycolysis, the overflow metabolism leads to the production of a surplus of pyruvate, which is then converted to lactate. This process results in the accumulation of intracellular lactate, which can potentially form La-CoA, an acyl group donor for the Kla of proteins.

**Figure 8:**
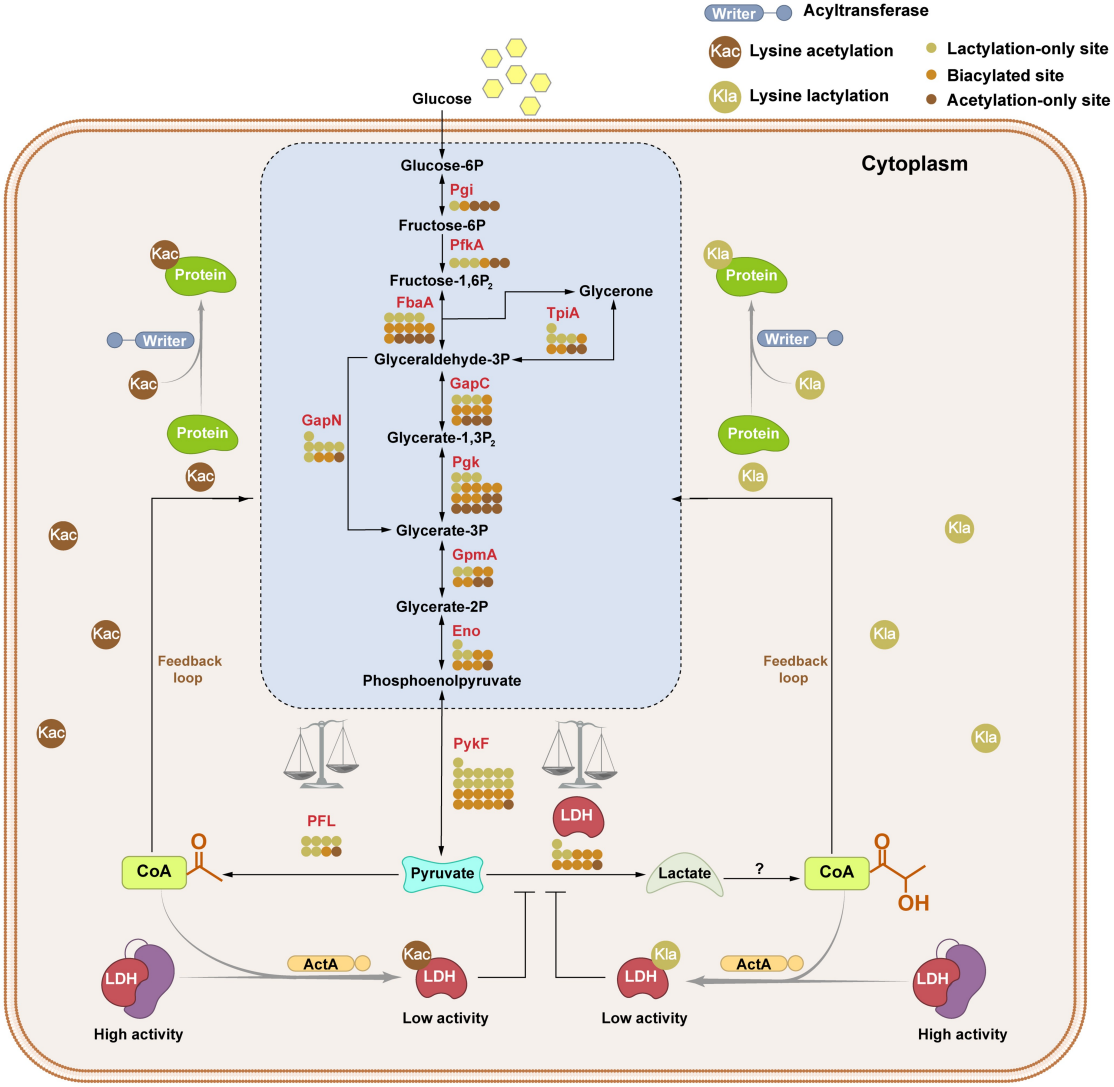
Regulatory diagram of lysine lactylation and acetylation in glycolytic pathway of *S. mutans* This schematic represents the glycolysis pathway in the cytoplasm of *S. mutans*, illustrating the sites of lysine lactylation (Kla) and acetylation (Kac) on glycolytic enzymes. These enzymes are denoted by their abbreviations, with circles indicating the sites of Kla or Kac. The dual-function acyltransferase ActA, which catalyzes both Kla and Kac on LDH and negatively regulates its enzymatic activity, is highlighted. The metabolism-PTM-metabolism feedback loop facilitates bacteria to fine-tune their metabolism through PTMs in response to the available metabolic intermediates. PTM, post-translational modification; Pgi, glucose-6-phosphate isomerase; PfkA, 6-phosphofructokinase; FbaA, fructose-bisphosphate aldolase; TpiA, triosephosphate isomerase; GapN, NADP-dependent glyceraldehyde-3-phosphate dehydrogenase; GapC, glyceraldehyde-3-phosphate dehydrogenase; Pgk, phosphoglycerate kinase; GpmA, 2,3-bisphosphoglycerate-dependent phosphoglycerate mutase; Eno, enolase; PykF, pyruvate kinase; LDH, lactate dehydrogenase; PFL, pyruvate-formate lyase.

Our study provides compelling evidence that among all lysine sites that exhibit both Kla and Kac in LDH, only the point mutation of K307 led to a significant reduction in their levels in an *in vitro* assay (Figure 6). In addition, the quantitative stoichiometry results from MS analysis showed that when LDH is incubated with ActA and lactyl-CoA or acetyl-CoA, K307 exhibits the highest levels of lactylation or acetylation compared to other lysine sites. These results highlight K307 as the primary lysine site that mediates both Kla and Kac in LDH. Indeed, further mutation of K307 to glutamine or arginine, which mimics the acylated or nonacylated states of LDH in *S. mutans*, alters the activity of LDH and subsequently influences lactate production and bacterial growth. The inhibitory effect of Kla and Kac on LDH activity may be due to electrostatic and conformational changes at K307, a critical lysine residue within the enzyme’s active domain (Figure S9). Kla and Kac introduce acyl groups that likely disrupt local charge and spatial configuration, impairing the binding affinity and catalytic activity of LDH. However, further investigation is required to fully elucidate these mechanisms. Overall, our study reveals that both Kla and Kac regulate the activities of metabolic enzymes and further modulate metabolic processes, suggesting the presence of a metabolism-PTM-metabolism feedback loop that enables the fine-tuning of metabolism depending on the available metabolic intermediates (Figure 8).

While this study provides significant insights into the crosstalk between Kla and Kac on LDH, some important questions remain unresolved. Beyond ActA, it is crucial to investigate whether other acyltransferases in *S. mutans* contribute to the regulation of Kla and Kac on LDH or other glycolytic enzymes. A systematic analysis of additional acyltransferases may reveal overlapping or complementary roles in regulating protein acylation. Furthermore, although we primarily focused on LDH due to its pivotal role in glycolysis, the observed crosstalk between Kla and Kac on other glycolytic enzymes, particularly those enriched near the glycolytic endpoint, warrants further exploration to understand how these modifications coordinate glycolytic flux and metabolic plasticity. Another intriguing avenue for future study is the potential interplay between nearby lysine residues on LDH. It is possible that spatial proximity facilitates cooperative or competitive crosstalk among these residues, further influencing enzymatic activity and regulation. Addressing these questions will deepen our understanding of the regulatory mechanisms underlying PTMs and their functional impact on bacterial metabolism.

In summary, our study elucidates the prokaryotic mechanism of metabolic regulation in *S. mutans* mediated by Kla and Kac, particularly in the glycolytic pathway. We demonstrated that the Kla and Kac of LDH dynamically change in response to metabolic intermediates, with ActA identified as the acyltransferase mediating these modifications and K307 as the principal lysine site regulating LDH activity, lactate production, and bacterial growth. These findings underscore the dynamic modulation of PTMs by metabolic intermediates, equipping *S. mutans* with a flexible mechanism to adapt to fluctuating environments. This work advances our understanding of protein lactylation and its crosstalk with acetylation, providing a foundation for future exploration of the dynamic interaction between the PTMs and metabolism in bacteria.

## Materials and methods

### Bacterial strains and growth conditions

*S. mutans* UA159 and its derivatives were obtained from our previous study and routinely cultured in BHI broth (Catalog No. 237500, BD Biosciences, Franklin Lakes, NJ) or BHI agar plates at 37°C under 5% CO_2_ [27]. *Escherichia coli* BL21 (DE3) and DH5α were purchased from the Tsingke Corporation (Beijing, China) and cultured in Luria-Bertani (LB) medium (Catalog No. 244620, BD Biosciences, Franklin Lakes, NJ) at 37°C with shaking at 200 r/min. The bacterial growth was monitored by measuring the OD_600_ using a spectrophotometer (Epoch, BioTek, Winooski, VT). All the bacterial strains and plasmids used in this study are listed in Table S6.

### Western blotting

Western blotting was performed as previously described [28]. For protein extraction, *S. mutans* and *E. coli* strains were harvested at the mid-log phase and lysed using RIPA buffer that contained a protease inhibitor cocktail. The protein concentrations were determined using a BCA protein assay kit (Catalog No. P0010, Beyotime Biotechnology, Shanghai, China) according to the manufacturer’s instructions. Equal amounts of protein (30 μg) were separated by 12% SDS-PAGE and transferred to polyvinylidene fluoride (PVDF) membranes (Catalog No. 1620177, Bio-Rad, Hercules, CA). The membranes were blocked in 5% non-fat milk for 1 h and then incubated with the primary pan-Kla (Catalog No. PTM-1401) and pan-Kac (Catalog No. PTM-102) antibodies (PTM BioLab, Hangzhou, China) overnight at 4°C. The membranes were washed and incubated with horseradish peroxidase (HRP)-conjugated secondary antibodies for 2 h at room temperature. The protein bands were visualized and captured with a Bio-Rad GS-700 imaging densitometer, and their intensities were quantified using ImageJ software (NIH, Bethesda, MD).

### Protein extraction and trypsin digestion

*S. mutans* UA159 strains in the mid-log phase were harvested by centrifugation at 4000 g for 15 min at 4°C, with three biological replicates prepared to ensure reliability. The bacterial pellets were resuspended in lysis buffer, followed by sonication on ice. After centrifugation at 20,000 g for 20 min at 4°C, the supernatant was collected, and the protein concentration was determined with the BCA protein assay kit. For digestion, the protein solution was reduced with 5 mM dithiothreitol for 30 min at 56°C and alkylated with 11 mM iodoacetamide for 15 min at room temperature in the dark. The protein sample was then diluted by adding 100 mM TEAB to a concentration of urea below 2 M. Finally, trypsin was added at a 1:50 trypsin-to-protein mass ratio for the first digestion overnight and a 1:100 trypsin-to-protein mass ratio for the second digestion for 4 h.

### Immunoaffinity enrichment

The digested peptides (2 mg) were desalted using a Strata X C18 SPE column (Phenomenex, Torrance, CA, USA) and vacuum dried. The Kla and Kac peptides were enriched by dissolving the tryptic peptides in NETN buffer (100 mM NaCl, 1 mM EDTA, 50 mM Tris-HCl, and 0.5% NP-40, pH 8.0) and incubating them with 30 µL of pre-washed antibody-conjugated protein A/G agarose beads (10 µg pan-Kla antibody, Catalog No. PTM-1401, and 10 µg pan-Kac antibody, Catalog No. PTM-102, both from PTM BioLab, Hangzhou, China) overnight with gentle shaking at 4°C. The beads were washed four times with NETN buffer and twice with ddH_2_O. The bound peptides were eluted from the beads with 0.1% trifluoroacetic acid. The eluted fractions were combined and vacuum dried. The resulting peptides were cleaned with C18 ZipTips (Catalog No. ZTC18M096, Millipore, Sigma, Burlington, MA) according to the manufacturer’s instructions followed by an HPLC-MS/MS analysis.

### HP-LC-MS/MS analysis

The tryptic peptides were dissolved in 0.1% formic acid (solvent A) and directly loaded onto a homemade reversed-phase analytical column (15 cm length, 75 μm i.d.). The gradient increased from 6% to 23% solvent B (0.1% formic acid in 98% acetonitrile) over 26 min, 23% to 35% in 8 min and increasing to 80% in 3 min and then held at 80% for the last 3 min, all at a constant flow rate of 400 nl/min on a nanoElute UHPLC system (Bruker Daltonics, Billerica, MA). The peptides were subjected to a capillary source and analyzed using a timsTOF Pro mass spectrometer (Bruker Daltonics, Billerica). The electrospray voltage applied was 1.60 kV. Precursors and fragments were analyzed at the TOF detector, with a MS/MS scan range from 100 to 1700 m/z. The timsTOF Pro was operated in parallel accumulation serial fragmentation (PASEF) mode. Precursors with charge states 0 to 5 were selected for fragmentation, and 10 PASEF-MS/MS scans were acquired per cycle. The dynamic exclusion was set to 30 s.

### Bioinformatic analysis

The resulting MS/MS data were processed using MaxQuant with an integrated Andromeda search engine (v1.5.2.8). Tandem mass spectra were searched against the Uniprot S. mutans UA159 database, concatenated with a reverse decoy database. Trypsin/P was specified as a cleavage enzyme that allowed up to four missing cleavages. The mass tolerance for the precursor ions was set as 20 ppm in the first search, and 5 ppm in the primary search, and the mass tolerance for fragment ions was set as 0.02 Da. Carbamidomethylation on Cys was specified as a fixed modification, and oxidation on Met, lactylation on Lys, and acetylation on the protein N-terminus were specified as variable modifications. The false discovery rate (FDR) thresholds for proteins, peptides, and modification sites were specified at 1%. The identified proteins were further subjected to GO and KEGG pathway analyses using the bioinformatics tool DAVID.

### Analysis of the protein-protein interaction networks

Protein-protein interaction networks (PPINs) were predicted using the STRING database with a combined score of > 0.4. The networks were visualized using Cytoscape with nodes representing proteins and edges representing protein interactions. Clusters in the network were identified using the Molecular Complex Detection (MCODE) app in Cytoscape.

### Glycolytic pH drop assay

The ability of *S. mutans* to lower the pH through glycolysis was assessed as previously described with some modifications [27]. To mimic the process of glycolysis, *S. mutans* were harvested from the mid-log phase cultures, washed twice, and resuspended in 50 mM potassium phosphate buffer that contained 50 mM KCl and 1 mM MgCl_2_, pH 7.2. Glucose was added to a final concentration of 1% to trigger the glycolytic process. The decrease in pH, which reflects the production of lactate, was monitored at regular intervals for up to 60 min using a pH meter. Aliquots were taken simultaneously at different intervals to subsequently assess lactate concentrations, LDH activity, acyl-CoA concentrations, and the levels of acylation.

### Purification of recombinant proteins

The corresponding genes were amplified from the *S. mutans* genomic DNA using PCR and cloned into *E. coli* expression vector pET28a. The constructed *E. coli* BL21 (DE3) cells transformed with *gene*-pET28a vectors were cultured in LB media containing kanamycin (Kana, 50 μg/ml) at 37°C and shaken until the cultures reached an OD_600_ of ∼ 0.6. The expression of proteins was induced with IPTG at 37°C for 6 h, and the cells were harvested by centrifugation. The cell pellets were resuspended in lysis buffer (20 mM Tris-HCl, pH 8.0, 500 mM NaCl, and 20 mM imidazole) and then lysed by sonication. The recombinant proteins were purified using a His-tagged protein purification kit (Catalog No. P2226, Beyotime Biotechnology, Shanghai, China) according to the manufacturer’s instructions and further desalted and concentrated using MWKO ultrafiltration (Catalog No. UFC9010 and UFC9003, MilliporeSigma, Burlington). The recombinant proteins were confirmed by SDS-PAGE (10%) and stored at −80°C for future use. All the primers used here are listed in Table S7.

### *In vitro* acylation assays

The *in vitro* acylation assays were performed as previously described with minor modifications [27,62]. Briefly, the purified LDH was incubated with La-CoA and Ac-CoA in acylation buffer (100 mM Tris-HCl, pH 7.5, 150 mM NaCl, 10 mM MgCl_2_, and 10% glycerol) with or without ActA or ActG at 37°C for 3 h. The reaction was terminated by adding 5× loading buffer and boiling for 10 min. The level of acylation of LDH was determined by western blotting with the pan-Kla and pan-Kac antibodies. In addition, the lactylated and acetylated bands were analyzed by LC-MS/MS.

### Assays of LDH activity

The activity of LDH was assayed by monitoring the reduction of NAD^+^ at 340 nm in a reaction mixture that contained 50 mM Tris-HCl (pH 7.5), 1 mM NADH, 1 mM pyruvate, and 100 nM purified LDH. The reaction was initiated by adding pyruvate, and the change in absorbance at 340 nm was monitored using a multimode microplate reader (Tecan Spark, Männedorf, Switzerland) for 5 min.

### Site-directed mutagenesis in *E. coli*

Primers designed to introduce mutations into the *ldh* gene were used for an overlap extension PCR. The mutated *ldh* and the linearized pET28a vector were then combined using an In-Fusion HD Cloning Kit (Catalog No. 639649, TaKaRa, Dalian, China). The resulting plasmid was transformed into *E. coli* Competent Cells DH5α and selected with kanamycin. After verification by sequencing, the confirmed plasmid was introduced into *E. coli* BL21 (DE3) to express the recombinant protein. All the primers used here are listed in Table S7.

### CRISPR-Cas9 mediated point mutation in *S. mutans*

The CRISPR-Cas9 system was used to generate the K307Q and K307R point mutations in the *ldh* gene from *S. mutans* [63]. Briefly, guide RNAs (gRNAs) were designed to target the specific *ldh* locus, and the pDL278 plasmid encoding gRNA was co-transformed into *S. mutans* along with a DNA repair template that contained the desired mutation, and the mutants were selected with spectinomycin (Spe, 100 μg/ml). The positive colonies were screened by PCR and confirmed by sequencing. All the primers used here are listed in Table S7.

### Growth curve analysis

Overnight cultures of *S. mutans* UA159 and its derivatives were diluted 1:100 into fresh TV media supplemented with 1% glucose and incubated at 37°C in 96-well plates, and each strain was added to three wells. Their growth was monitored by measuring the OD_600_ using a spectrophotometer at 1 h intervals over a 16 h period. The growth curves were plotted to compare the growth dynamics of the different strains.

### Molecular dynamics simulation

Molecular dynamics (MD) simulations were performed using GROMACS (gmx2020.6_GPU) with the AMBER99SB-ILDN force field for the protein and TIP3P water model for solvation. The lysine at position 307 of LDH was mutated to arginine (K307R) and glutamine (K307Q) using the Mutagenesis plugin in PyMOL (v2.5.5). The protein complex was solvated in a cubic box with TIP3P water molecules, and Na^+^ and Cl^−^ ions were added to neutralize the system. Energy minimization was conducted in two stages: first, using the steepest descent algorithm, and then applying the Particle-Mesh Ewald (PME) method for long-range electrostatic interactions. The system was equilibrated in two phases: first under constant volume (NVT) to stabilize the temperature at 300 K, and then under constant pressure (NPT) at 1 bar. After equilibration, a production MD simulation was run for 10,000 ps (10 ns) to study the dynamics of the mutated proteins at 300 K and 1 bar. Post-simulation analysis was conducted to assess the stability and structural changes, using VMD and PyMOL to visualize the protein conformations and calculate the root mean square deviation (RMSD) values to evaluate the effects of the mutations.

### Statistical analysis

Statistical analyses were performed using GraphPad Prism 9.0 (GraphPad Software, San Diego, CA). All the experimental data were presented as the mean ± standard deviation (SD) of at least three independent biological replicates. Differences between the groups were analyzed using a one-way analysis of variance (ANOVA) followed by a post-hoc Tukey’s test for multiple comparisons or using a two-tailed Student’s *t*-test when only two groups were compared. The growth curve data were analyzed by a two-way ANOVA with repeated measures followed by Bonferroni post-tests. *P* < 0.05 were considered statistically significant.

## Supplementary Material

qzaf073_Supplementary_Data

## Data Availability

The raw proteomic data reported in this study have been deposited in the Open Archive for Miscellaneous Data [64] at the National Center for Bioinformation (NGDC), China National Center for Bioinformation (CNCB) (OMIX: 011420), which are publicly accessible at https://ngdc.cncb.ac.cn/omix. All other data generated or analyzed during this study are included in the Supplementary material.

## CRediT author statement

**Qizhao Ma:** Methodology, Software, Validation, Formal analysis, Investigation, Resources, Data curation, Writing – Original draft, Visualization, Project administration, Funding acquisition. **Tao Hu:** Conceptualization, Resources, Data curation, Writing – review & editing, visualization. **Yongwang Lin:** Software, Validation, Formal analysis, Data curation. **Jing Li:** Investigation, Resources. **Jun Huang:** Validation, Formal analysis. **Qiong Zhang:** Data curation, Writing – review & editing, Funding acquisition. **Tao Gong:** Investigation, Resources, Funding acquisition. **Xuedong Zhou:** Resources, Supervision, Funding acquisition. **Lei Lei:** Conceptualization, Writing – review & editing, Supervision, Project administration. **Jing Zou:** Conceptualization, Methodology, Writing – review & editing, Supervision, Funding acquisition. **Yuqing Li:** Conceptualization, Writing – review & editing, Supervision, Funding acquisition. All authors have read and approved the final manuscript.
