# Supplementary material for: Crosstalk Between Lysine Lactylation and Acetylation Regulates Lactate Dehydrogenase in Streptococcus mutans
Source: Genomics Proteomics Bioinformatics. 2025 Aug 22;23(6):qzaf073. doi: 10.1093/gpbjnl/qzaf073 (PMC13221243; doi:10.1093/gpbjnl/qzaf073)
Supplement: qzaf073_Supplementary_Data [file qzaf073_supplementary_data.zip › Supplementary material captions.docx]

**S****upplementary materials**

**Figure S****1 Detection of lysine lactylation and acetylation in *Streptococcus mutans* and *Escherichia coli***

Coomassie staining and western blotting were performed with whole cell lysates of *S. mutans* and *E. coli* using pan-Kla and pan-Kac antibodies to visualize the amounts of proteins (**A**), the presence of Kla (**B**) and Kac (**C**) in these bacterial strains. Kla, lysine lactylation; Kac, lysine acetylation.

**Figure S2 Protein-protein interaction networks and KEGG pathway enrichment analysis of proteins with coexisting lysine lactylation and acetylation sites**

**A**. Protein-protein interaction network of proteins with more than six coexisting Kla and Kac sites, analyzed using the MCODE plug-in toolkit in the Cytoscape software. **B**. KEGG pathway enrichment analysis of proteins with more than six coexisting Kla and Kac sites. Kla, lysine lactylation; Kac, lysine acetylation.

**Figure S3 Glycolytic pH drop assay in *S. mutans***

The pH value of the supernatant of *S. mutans* grown in the presence of 1% glucose over 60 min.

**Figure S4 Detection of lysine lactylation and acetylation levels of LDH in *S. mutans* UA159/pDL278 and UA159/pDL278-*actA***

Protein samples were extracted from the wild-type strain harboring the empty vector (UA159/pDL278) and the *actA* overexpression strain (UA159/pDL278-*actA*). Western blotting was performed to analyze the lysine lactylation and acetylation levels of LDH in these samples. Kla, lysine lactylation; Kac, lysine acetylation; LDH, lactate dehydrogenase; Gyrase, loading control.

**Figure S5 SDS-PAGE analysis of recombinant proteins LDH and ActA**

The gel image presents the results of SDA-PAGE used to evaluate the purity of lactate dehydrogenase (LDH) and the acyltransferase ActA. The left and right lanes show the protein ladder with molecular weights indicated.

**Figure S****6 MS/MS spectrum of lactylated peptide from common Kla and Kac sites in LDH**

The identified lactylated peptides are LDLVGK(la)NLAINK, FSGFPAEK(1a)VIGSGTSLDTAR, QALAEK(1a)LDVDAR, K(1a)GATFYGIAVALAR, PVNIPLNDAEK(1a)QK, ELK(1a)AIIDEAFSK, and AIIDEAFSK(1a)EEFAAAAR, respectively.

**Figure S****7 MS/MS spectrum of acetylated peptide from common Kla and Kac sites in LDH**

The identified acetylated peptides are LDLVGK(ac)NLAINK, FSGFPAEK(ac)VIGSGTSLDTAR, QALAEK(ac)LDVDAR, K(ac)GATFYGIAVALAR, PVNIPLNDAEK(ac)QK, ELK(ac)AIIDEAFSK, and AIIDEAFSK(ac)EEFAAAAR, respectively.

**Figure S8 SDS-PAGE analysis of the recombinant LDH with point mutations**

The gel image displays the SDS-PAGE results for various mutant forms of LDH. The first gel on the left shows LDH mutants where specific lysine (K) residues have been substituted with glutamine (Q). The second gel on the right displays LDH mutants with lysine residues substituted with arginine (R). The molecular weight markers are indicated on the left of each gel.

**Figure S9 Structural analysis of LDH with K307 mutations to arginine and glutamine**

Structural comparison of wild-type LDH and the mutants K307R (lysine to arginine) and K307Q (lysine to glutamine) using molecular dynamics simulations. The mutations were modeled using PyMOL, and molecular dynamics simulations were performed with GROMACS software. The structural changes due to these mutations were analyzed by calculating the root mean square deviation (RMSD) values to assess the impact on protein conformation. The RMSD values indicate the extent of structural alterations caused by the mutations: LDH 307K to R (RMSD = 0.936, panel A) and LDH 307K to Q (RMSD = 1.029, panel B). The larger the RMSD, the greater the structural disruption caused by the mutations. The structural changes likely contribute to the functional alterations of LDH activity, which may be regulated by acetylation and lactylation at the K307 site.

**Table S1 List of all identified lactylated and acetylated proteins and sites in *S. mutans***

**Table S****2 GO enrichm****ent analysis of proteins with Kla and Kac sites**

**Table S****3 KEGG pathway analysis of proteins with Kla and Kac sites**

**Table S4 Protein-protein interaction networks of proteins with common Kla and Kac sites**

**Table S5 List of identified Kla and Kac sites of LDH *in vitro* acylation assays**

**Table S6 Bacterial strains and plasmids used in this study**

**Table S7 All primers used in this study**
