# Supplementary figures and images for: Crosstalk Between Lysine Lactylation and Acetylation Regulates Lactate Dehydrogenase in Streptococcus mutans
Source: Genomics Proteomics Bioinformatics. 2025 Aug 22;23(6):qzaf073. doi: 10.1093/gpbjnl/qzaf073 (PMC13221243; doi:10.1093/gpbjnl/qzaf073)

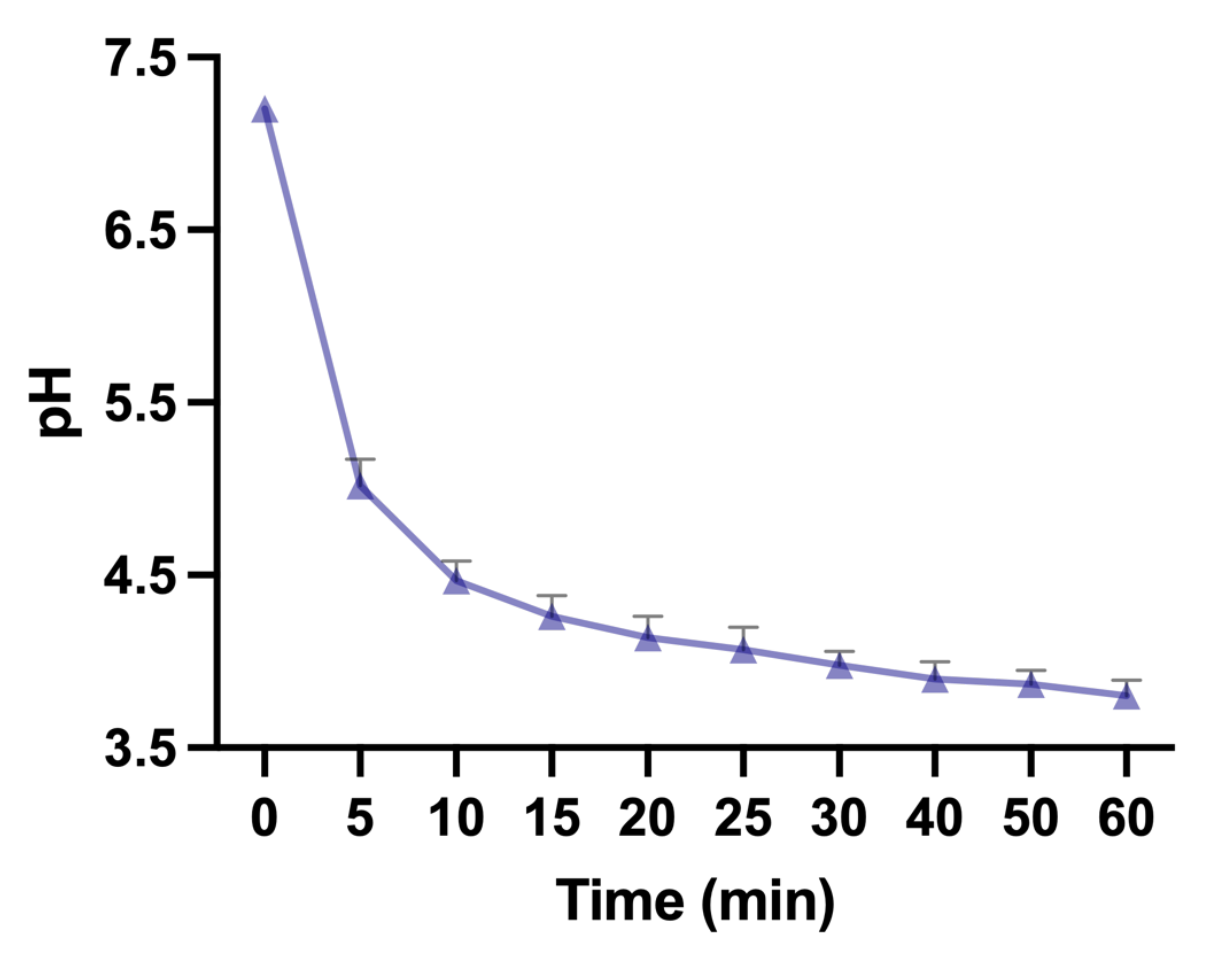

Supplement: qzaf073_Supplementary_Data [file qzaf073_supplementary_data.zip › Figure S3.tif]

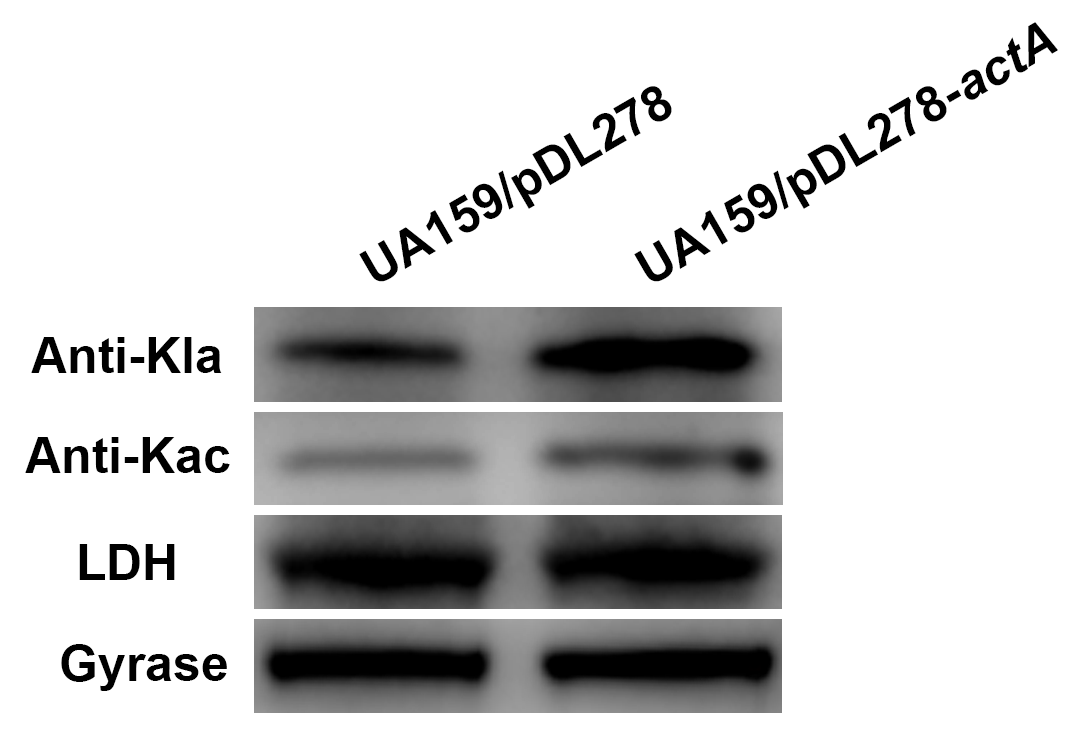

Supplement: qzaf073_Supplementary_Data [file qzaf073_supplementary_data.zip › Figure S4.tif]

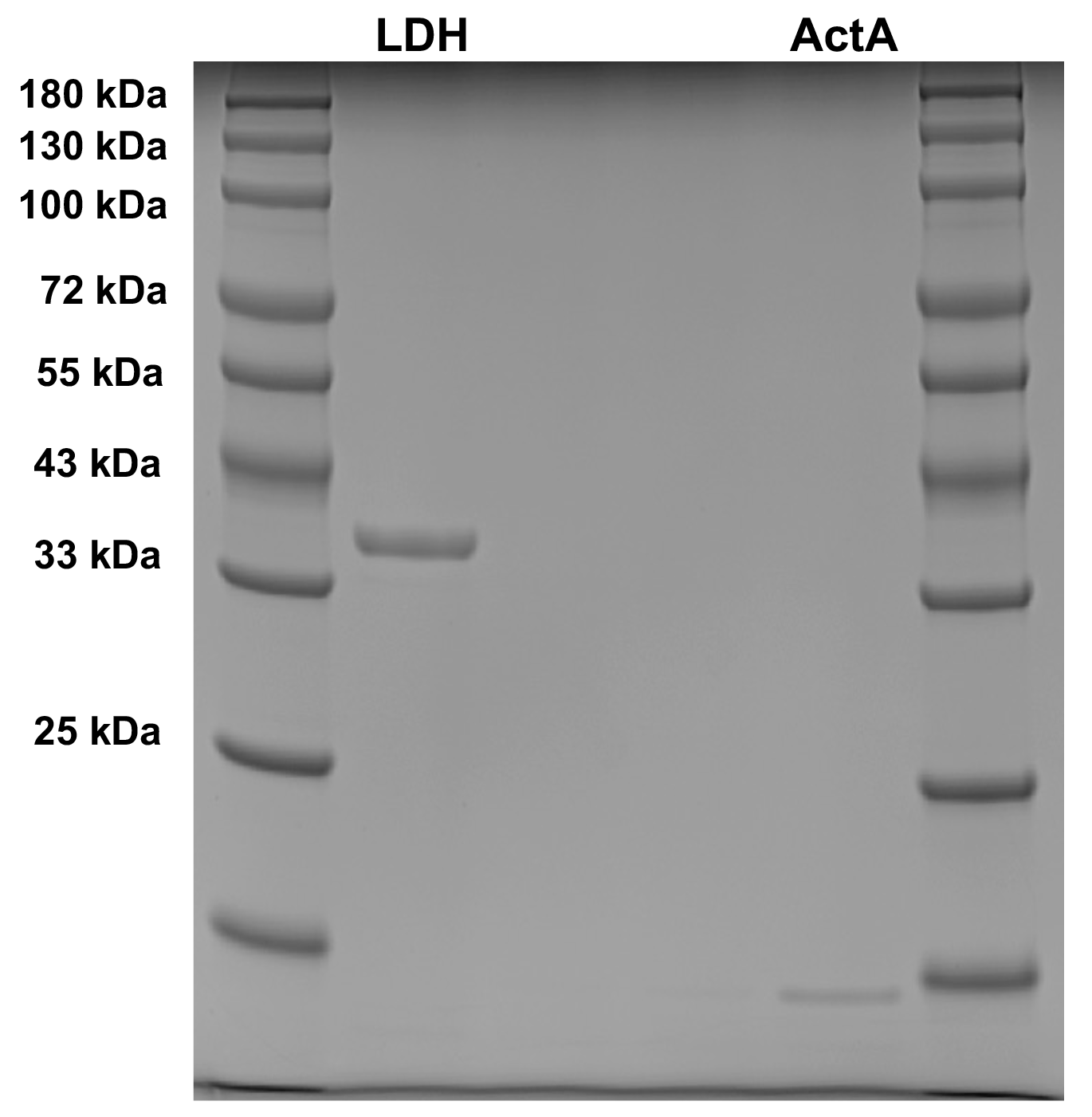

Supplement: qzaf073_Supplementary_Data [file qzaf073_supplementary_data.zip › Figure S5.tif]

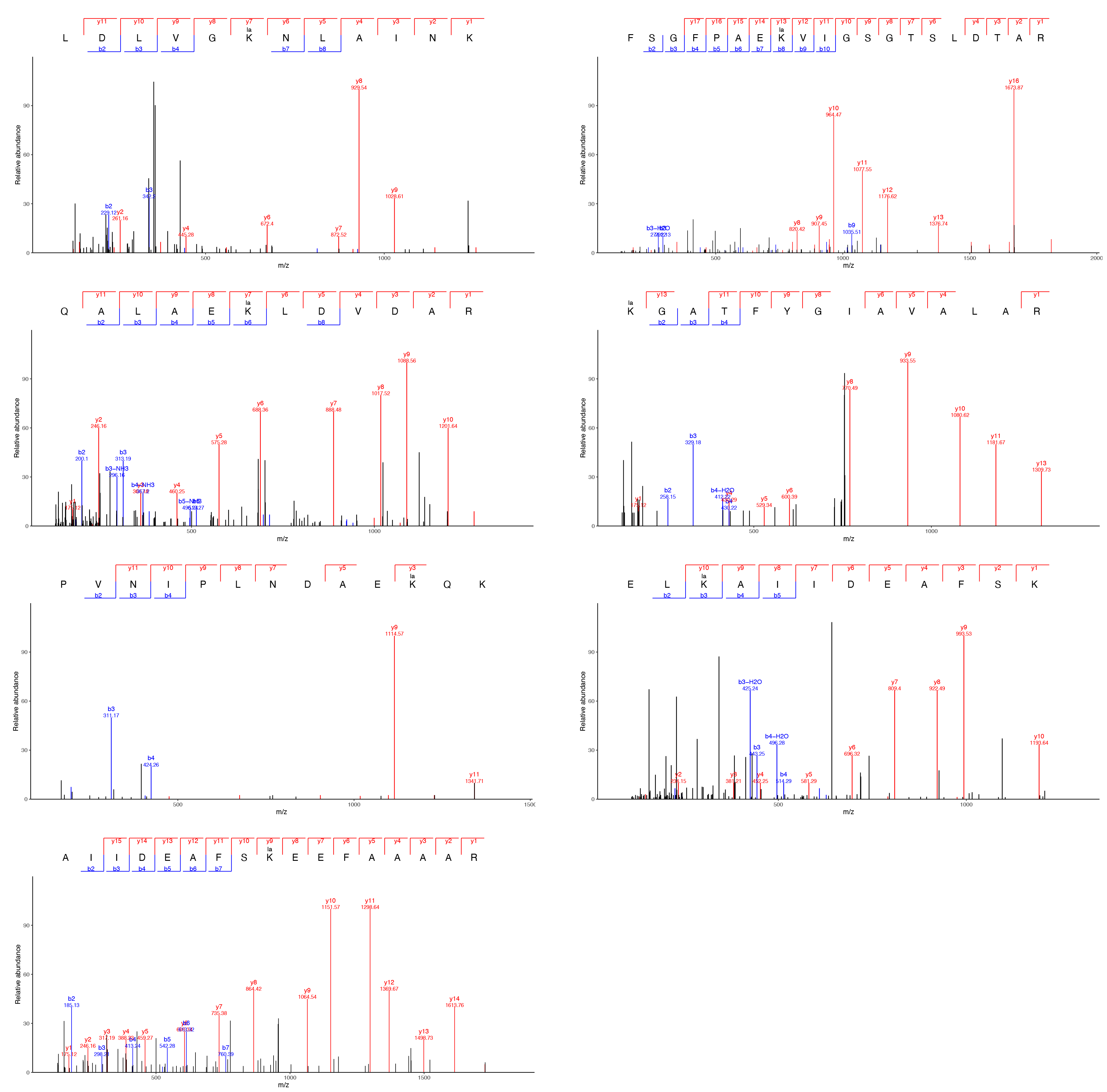

Supplement: qzaf073_Supplementary_Data [file qzaf073_supplementary_data.zip › Figure S6.tif]

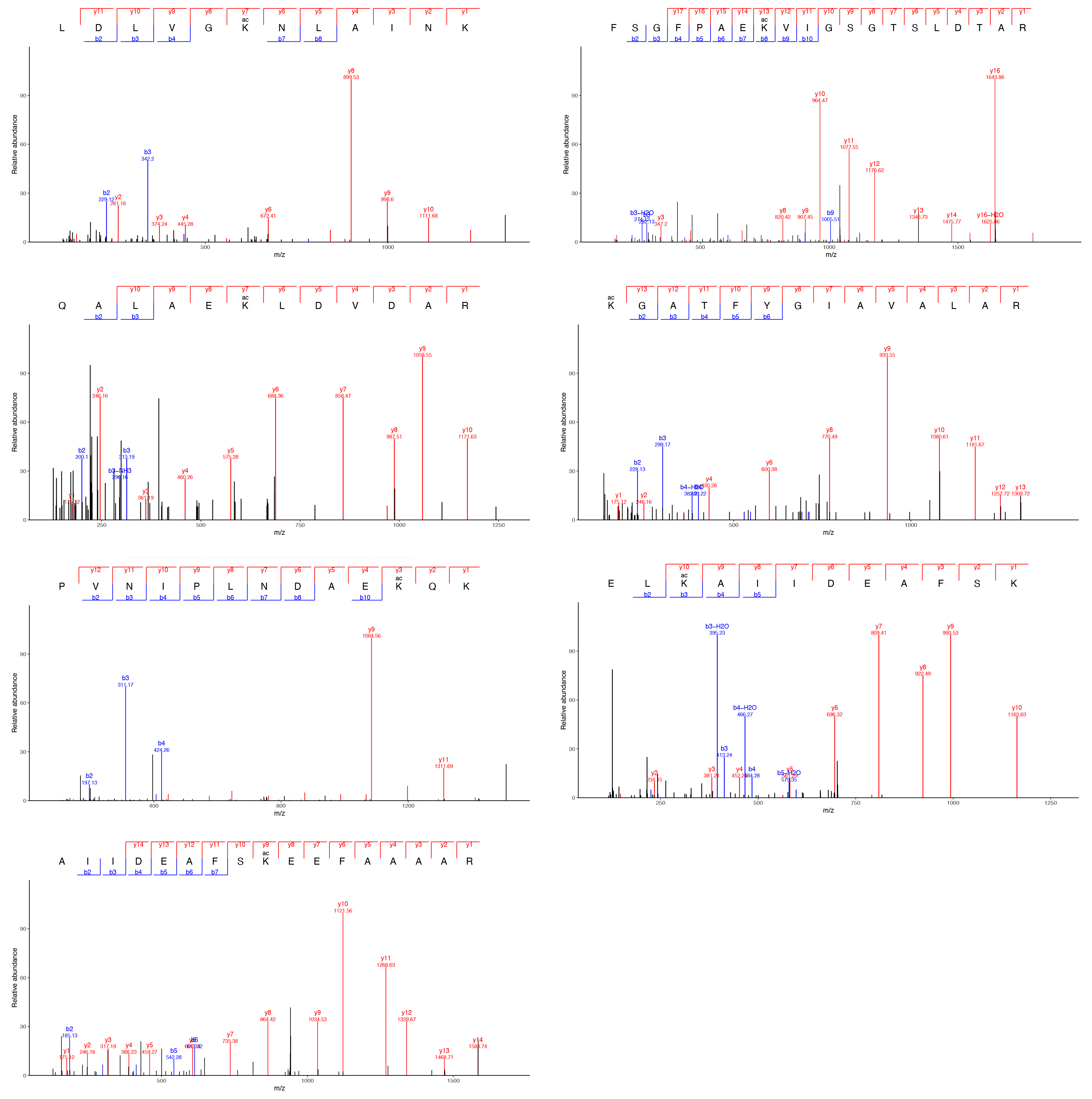

Supplement: qzaf073_Supplementary_Data [file qzaf073_supplementary_data.zip › Figure S7.tif]

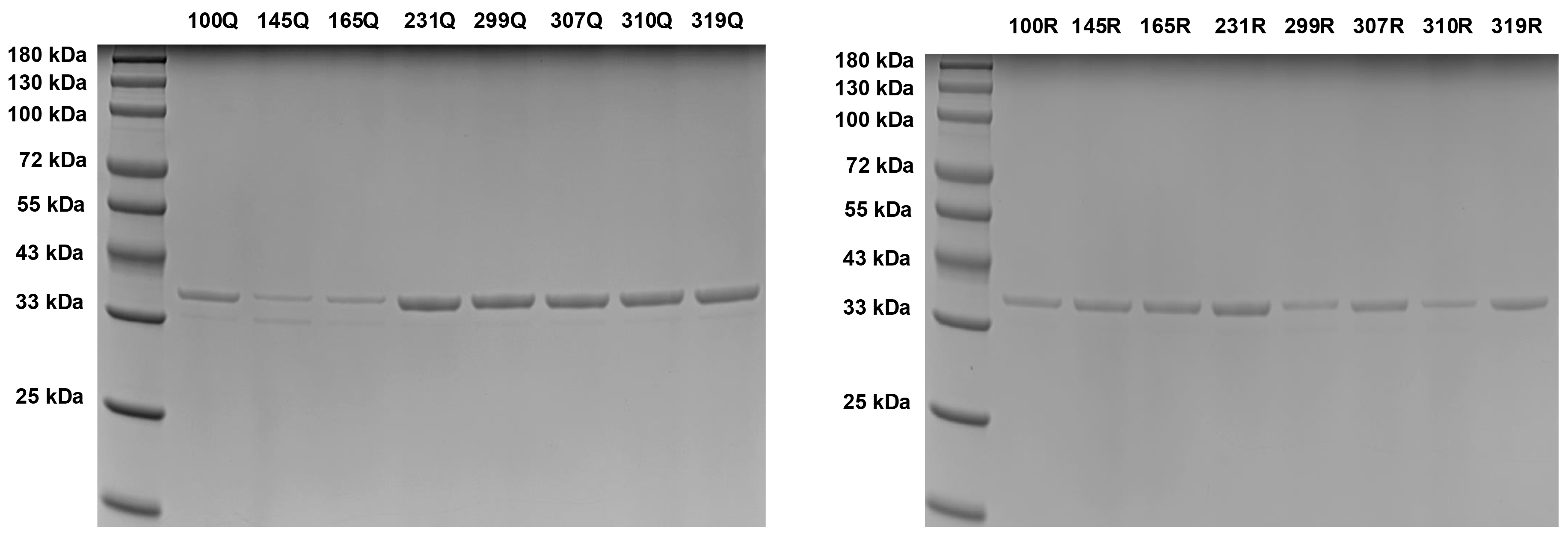

Supplement: qzaf073_Supplementary_Data [file qzaf073_supplementary_data.zip › Figure S8.tif]

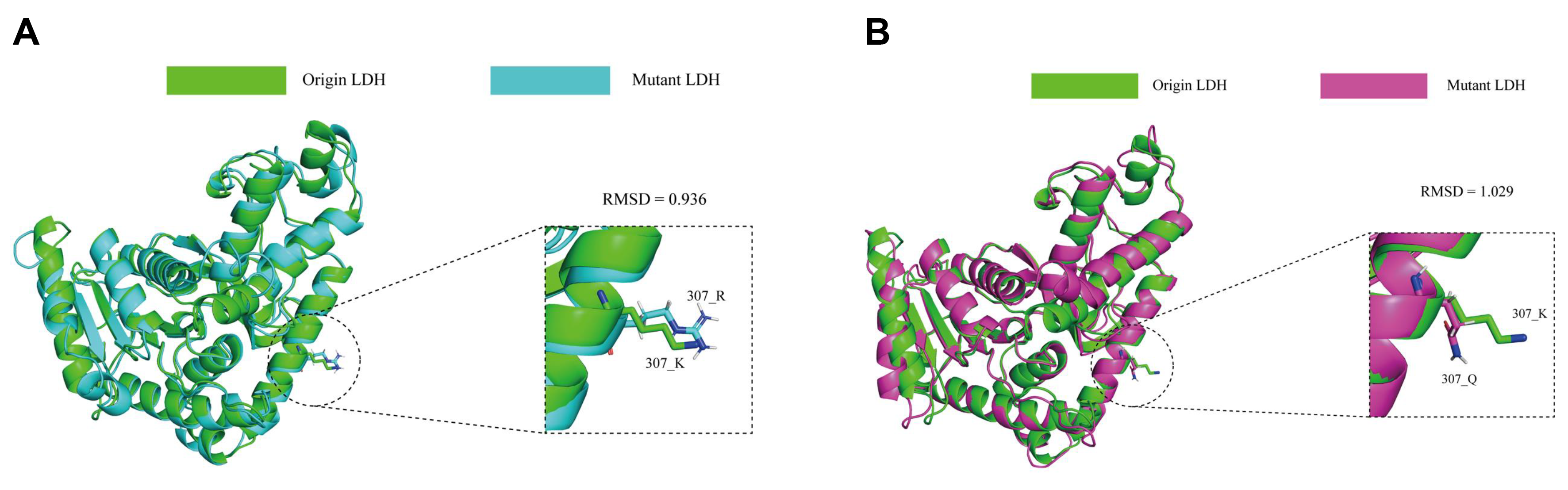

Supplement: qzaf073_Supplementary_Data [file qzaf073_supplementary_data.zip › Figure S9.tif]

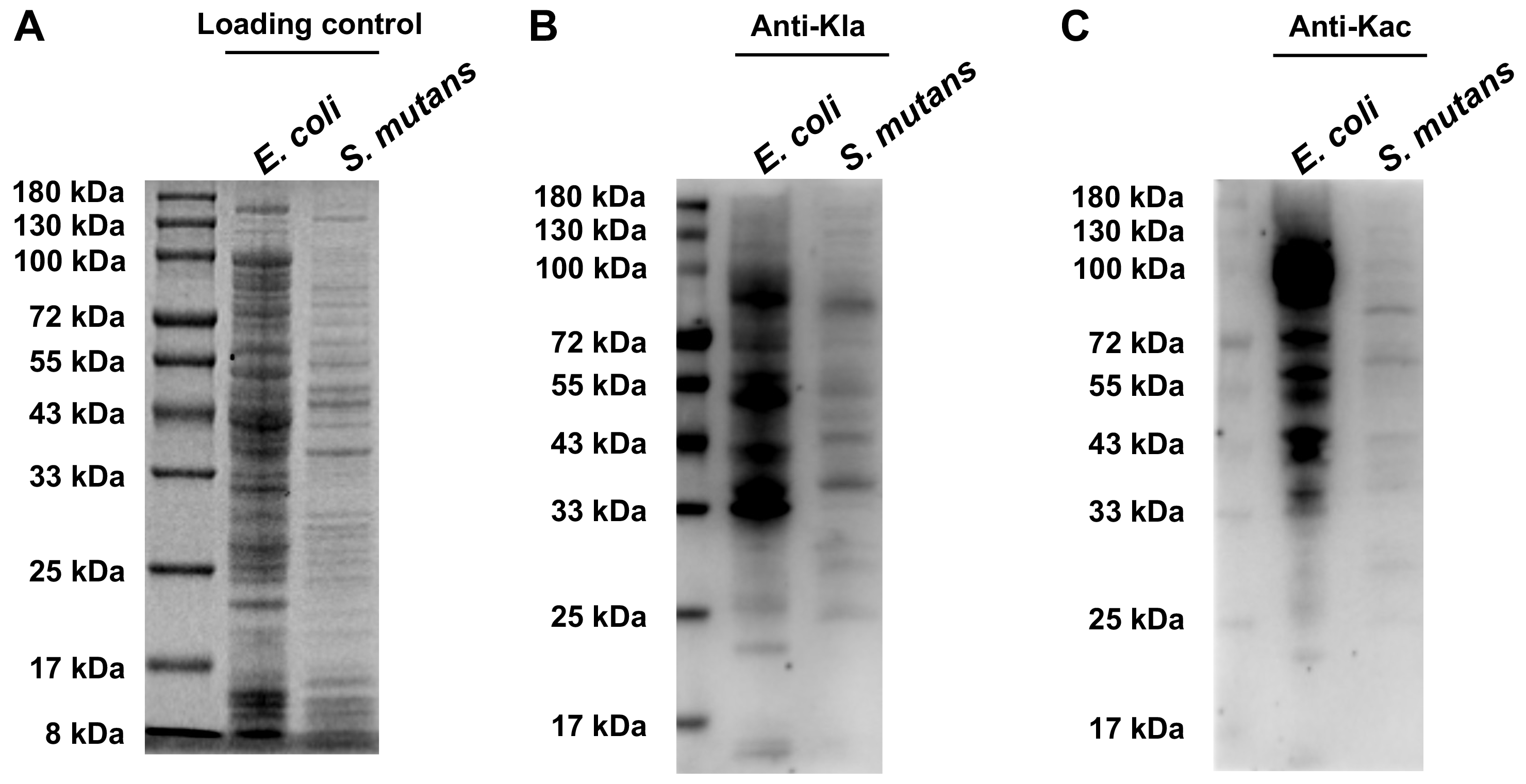

Supplement: qzaf073_Supplementary_Data [file qzaf073_supplementary_data.zip › Figure S1.tif]

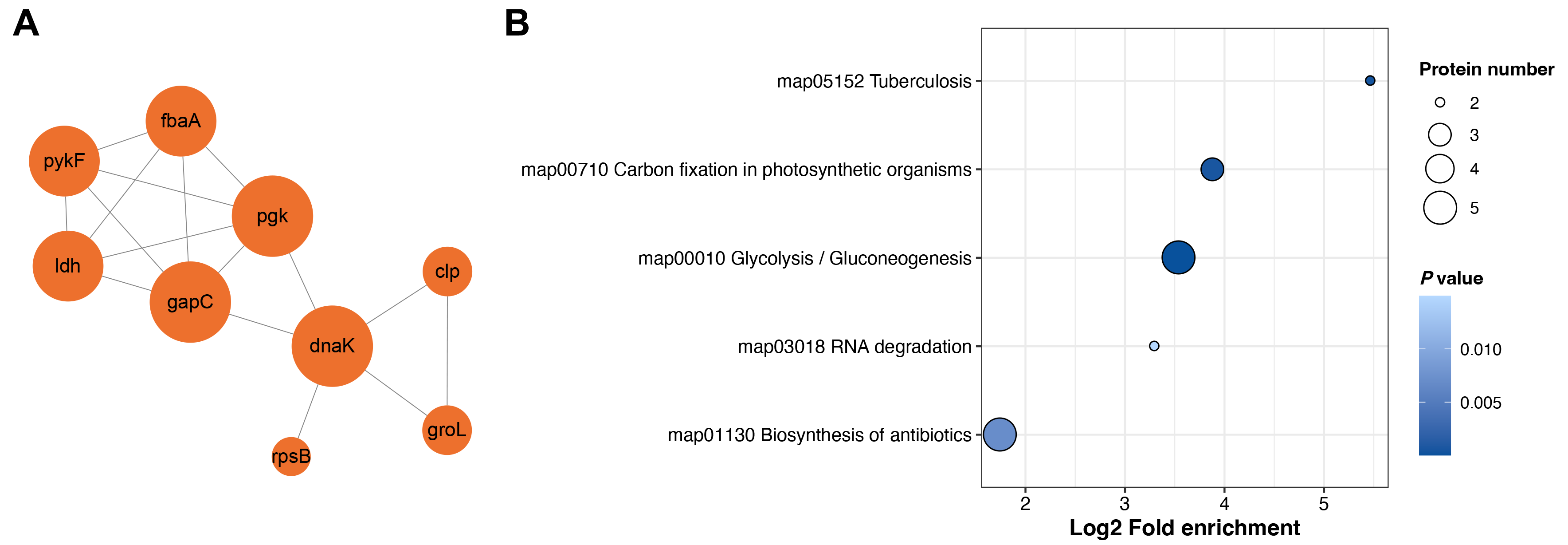

Supplement: qzaf073_Supplementary_Data [file qzaf073_supplementary_data.zip › Figure S2.tif]
